# Fatigue Limit Doubling in High‐Strength Martensitic Steel through Crack Embryo Engineering–Cyclic‐Training‐Driven Self‐Optimization

**DOI:** 10.1002/advs.202504165

**Published:** 2025-06-29

**Authors:** Kazuho Okada, Kaneaki Tsuzaki, Eri Nakagawa, Akinobu Shibata

**Affiliations:** ^1^ Research Center of Structural Materials National Institute for Materials Science (NIMS) Tsukuba 305‐0047 Japan

**Keywords:** crack initiation, elastic anisotropy, fatigue limit, martensitic steel, plastic incompatibility, self‐optimization, training

## Abstract

Achieving superior fracture resistance under cyclic loading–specifically, a high‐fatigue limit–is crucial for ensuring structural safety and supporting a sustainable society. This study demonstrates a breakthrough in overcoming the conventional fatigue limit ceiling in high‐strength as‐quenched martensitic steel by enhancing resistance to crack initiation. In the as‐heat‐treated state, high‐angle boundaries with large elastic misfits and plastic incompatibility served as precursory sites for intrusions/extrusions (these are defined as “crack embryos”), eventually leading to fatigue crack initiation. Remarkably, after the pre‐fatigue training, surface crack initiation is entirely suppressed, doubling the fatigue limit with minimal change in tensile strength. A novel concept of “crack embryo engineering” is introduced, which targets the prevention of crack embryo formation by extracting intrinsic microstructural self‐optimization against fatigue deformation: macroscopic hardness homogenization and selective nano‐hardening of the precursory sites. This self‐optimization strategy offers a versatile approach to improving fatigue limit in general steels, providing an effective alternative to tempering heat treatment that inevitably sacrifices tensile strength.

## Graphical abstract

This study demonstrates a breakthrough in overcoming the conventional fatigue limit ceiling in high‐strength as‐quenched martensitic steel; maximum stress corresponding to the fatigue limit significantly increased from 675 to 1300 MPa with minimal sacrifice in tensile strength (1.5–1.6 GPa). The authors find that cyclic deformation, conventionally considered harmful, intrinsically possesses microstructural self‐optimization effects, dramatically enhancing crack initiation resistance.

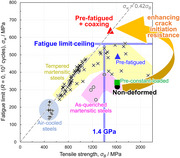

## 1 Introduction

**Figure 1:**
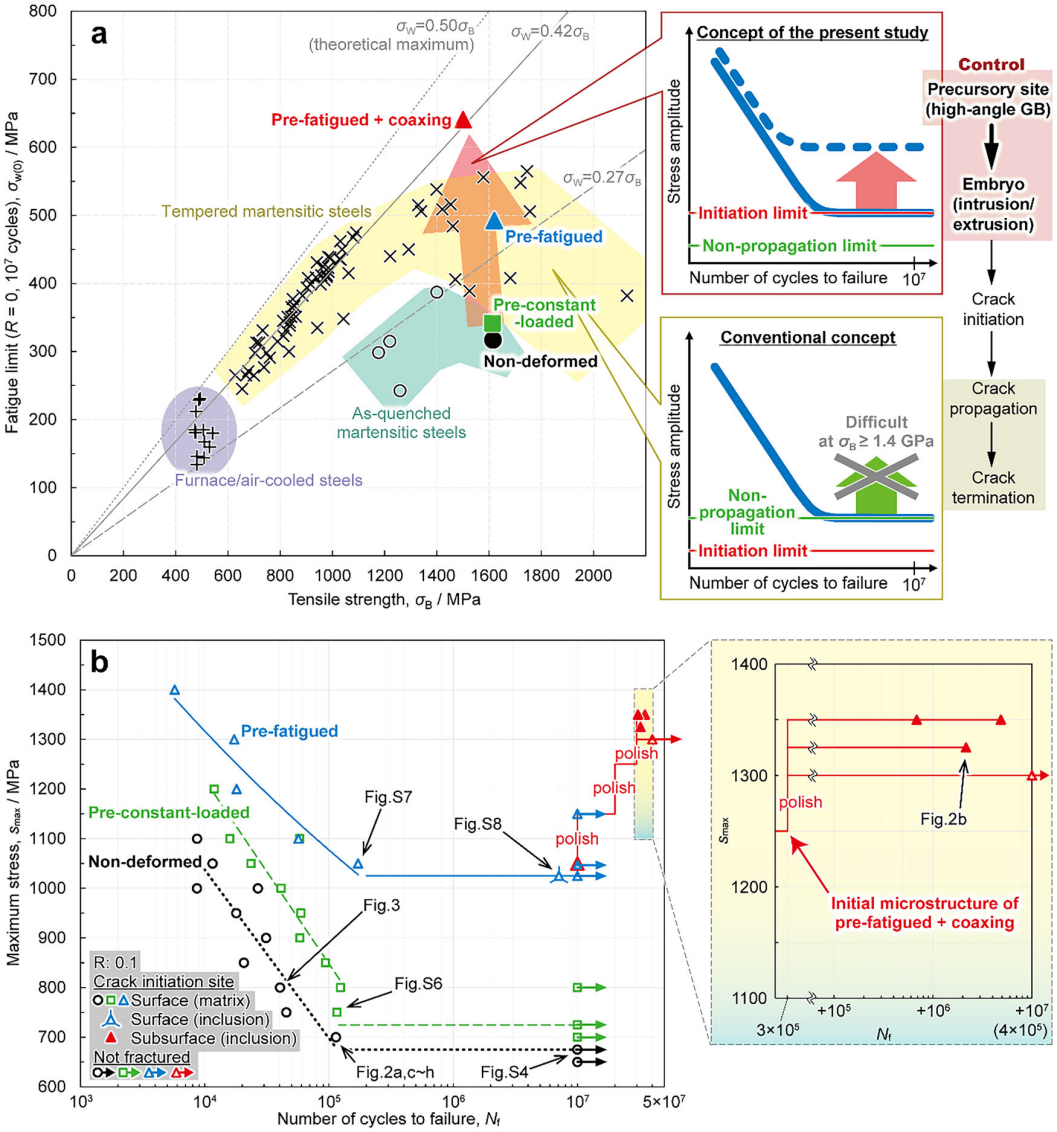
Significant improvement of fatigue limit by suppressing crack initiation via pre‐fatigue deformation in as‐quenched martensitic steels. a) Relationship between tensile strength (*σ*_B_) and 10^7^ cycles fatigue limit at a stress ratio of 0 (*σ*_W(0)_, expressed as stress amplitude). Solid black circles, green squares, and blue and red triangles indicate the non‐deformed, pre‐constant‐loaded, pre‐fatigued, and pre‐fatigued + coaxing specimens, respectively. Furnace/air‐cooled steels^[^ ^7^ ^]^ (plus mark), tempered martensitic steels^[^ ^7c^ ^–^ ^9^ ^]^ (cross mark), and as‐quenched martensitic steels^[^ ^24^, ^25^, ^30^, ^31^ ^]^ (open black mark) are cited from the open‐source database and literature. Contrary to the conventional concept that *σ*_W_ corresponds to the crack non‐propagation limit, the present study aimed at improving *σ*_W_ by controlling the stages before intrusion/extrusion (crack embryo) formation and improving the crack initiation limit. b) Number of cycles to failure against maximum stress (*s*_max_) in the fatigue test at *R* = 0.1, in which the crack initiation site for each test is indicated with specific symbols: at the surface matrix (open mark), surface inclusion (open triangle with extended corners), subsurface inclusion (solid triangle), or non‐fractured (open mark with arrow). The *s*_max_ corresponding to the fatigue limit (*s*_max‐W_) is converted to *σ*_W(0)_ using the modified Goodman diagram and plotted in (a).

To achieve a safe and sustainable society, the durability and reliability of metallic components, in addition to high‐strength, are crucial across various industries—from aerospace and automotive to infrastructure and manufacturing. Cyclic loading can cause catastrophic failures at much lower stresses than those under monotonic loading, known as fatigue fracture.^[^ ^1^, ^2^ ^]^ Safety and reliability alone would suggest making metallic components thicker and heavier. However, thin and lightweight components are essential for adopting environmentally friendly solutions through energy savings and reducing carbon emissions in such industries as automotive. To simultaneously meet these demands, establishing design guidelines for materials with high fatigue resistance is crucial.^[^ ^3^, ^4^, ^5^, ^6^ ^]^ As shown in **Figure** **1**a, the fatigue limit (*σ*_W_, expressed by stress amplitude) of steel increases proportionally with tensile strength (*σ*_B_), following *σ*_W_ = 0.42*σ*_B_ at a stress ratio (*R*) of 0 for furnace/air‐cooled steels^[^ ^7^ ^]^ (plus marks) and tempered martensitic steels^[^ ^7^, ^8^, ^9^ ^]^ (cross marks). However, when *σ*_B_ exceeds ≈1.4 GPa, further increases in *σ*_B_ do not improve or rather decrease *σ*_W_, that is, the “fatigue limit ceiling”. Consequently, the practical application of ultra‐high‐strength steel is restricted.^[^ ^10^ ^]^

Generally, *σ*_W_ equals the higher of the “crack initiation limit” and “crack non‐propagation limit”. The crack initiation limit is defined as the maximum stress below which no cracks initiate. On the other hand, the crack non‐propagation limit is the maximum stress where cracks can initiate but do not propagate to final rupture. For most steels, the ceiling of the non‐propagation limit becomes that of *σ*_W_ since the non‐propagation limit is higher than the initiation limit. Extensive research on crack propagation/termination behaviors has demonstrated the quantitative correlation between the non‐propagation limit and the macroscopic mechanical properties, such as hardness and tensile/yield strength.^[^ ^11^, ^12^, ^13^, ^14^, ^15^, ^16^ ^]^ These properties correlate positively with the non‐propagation limit because the hardness/strength of the crack‐front matrix is one of the controlling factors in crack‐termination. These results indicate that the upper limit size of the non‐propagation crack, necessary for achieving the *σ*_W_–*σ*_B_ proportional relationship, decreases with increasing the *σ*_B_ and becomes submicron size when *σ*_B_ > ≈1.4 GPa (see “Upper limit size of non‐propagation crack” and Figure S1, supporting information). Terminating submicron cracks is challenging since there is insufficient prior plastic deformation for crack closure.^[^ ^17^ ^]^ This indicates an intrinsic ceiling of the non‐propagation limit, while the theoretical ceiling of the crack initiation limit has not been reported. Thus, improving the crack initiation limit holds more potential to overcome the fatigue limit ceiling.^[^ ^18^, ^19^ ^]^ However, a systematic framework for designing materials with superior crack initiation resistance has been lacking. To date, conventional research on structural metals and alloys has primarily focused on the balance between tensile strength and elongation,^[^ ^20^, ^21^, ^22^ ^]^ and the tensile properties (bulk average properties) are closely related to the non‐propagation limit because crack termination can occur throughout a material.^[^ ^11^, ^12^, ^13^, ^14^, ^15^, ^16^ ^]^ In contrast, crack initiation occurs at the weakest domain in a material, which cannot be evaluated from the bulk average properties and is highly microstructure‐dependent; thus, designing crack‐initiation‐resistant material holds an immense potential to utilize the microstructure.

It is well known that shot peening can improve the crack initiation limit.^[^ ^23^ ^]^ The compressive residual stress, introduced in the near‐surface layer, substantially reduces the effective stress during fatigue loading and suppresses crack initiation at the surface. However, in as‐quenched martensitic steel, vital for high‐strength low/medium‐carbon steels, shot peening merely shifts the crack initiation site from surface matrix to subsurface matrix (not to inclusion), with only a slight improvement in the *σ*_W_: from *σ*_W_ = 0.25*σ*_B_ to 0.26*σ*_B_.^[^ ^24^ ^]^ This would be due to the stress gradient introduced by shot peening from the surface to the interior acting as a new weak domain. A novel microstructure design strategy for improving crack initiation resistance throughout the sample from surface to subsurface is crucial.

Due to the low fracture resistance of as‐quenched martensitic steels, tempered martensitic steels with sacrificed *σ*_B_ are applied for structural components. Wider application of as‐quenched martensitic steels in their highest strength would contribute to a sustainable society. In martensitic steels, the fatigue cracks initiate from intrusions/extrusions formed along specific grain boundaries (GBs).^[^ ^25^, ^26^ ^]^ While intrusions/extrusions have not yet become cracks, the local stress concentration caused by surface roughness promotes local plastic deformation, leading to crack initiation. Therefore, we newly defined intrusions/extrusions as “crack embryos”. Controlling the formation of crack embryos would suppress crack initiation. However, it remains unclear why localized plastic deformation forms intrusions/extrusions at specific GBs in martensitic structures. In the submicron/nano‐crystalline materials, the intrusions/extrusions do not originate from persistent slip bands (PSBs) because the grain size is too small for the dislocation activity leading to PSB.^[^ ^27^, ^28^ ^]^

This study demonstrates a fatigue limit doubling, overcoming the conventional fatigue limit ceiling, in high‐strength as‐quenched martensitic steel by improving crack initiation resistance throughout the sample from surface to subsurface via pre‐fatigue training. Additionally, we elucidated the mechanism of i) crack initiation and ii) suppression of crack initiation in pre‐fatigued specimens. Specifically, we identified sites that could potentially form intrusions/extrusions, that is “precursory sites of crack embryos”, and demonstrated successful strategies for deactivating them, introducing “crack embryo engineering” as a novel design concept for superior fatigue resistance. As shown on the right side of Figure 1a, conventional studies have primarily focused on crack propagation/termination, lacking a framework to thoroughly understand and suppress crack initiation. The crack embryo engineering newly subdivides the crack initiation process into “precursory site → embryo → crack” and aims to suppress crack initiation by systematically understanding and controlling the stages before crack embryo formation, unlocking a new potential of the microstructure. This study specifically targets the fatigue limit at 10^7^ cycles though the very‐high‐cycle fatigue property is also an important topic.

## 2 Results and Discussion

### 2.1 Significant Improvement of Fatigue Limit by Pre‐Fatigue Deformation

In this study, non‐deformed specimens (Fe‐3Mn‐0.2C (wt.%)) in the as‐quenched condition served as the base material, along with three specimens subjected to different pre‐deformations: pre‐constant‐loaded, pre‐fatigued, and pre‐fatigued + coaxing. The non‐deformed specimen exhibited *σ*_B_ and 0.2% proof stress (*σ*_0.2_) of 1615 and 1118 MPa, respectively. In the pre‐deformations, the maximum stress (*s*_max_) for both the pre‐constant‐loaded and pre‐fatigued specimens was 1000 MPa, but the stress amplitude differed: 0 MPa for the pre‐constant‐loaded and 50 MPa for the pre‐fatigued. Figure 1b shows the number of cycles to failure against *s*_max_ in the fatigue test at *R* = 0.1, with crack initiation sites marked by specific symbols. The *s*_max_ at the fatigue limit (*s*_max‐W_) was 675, 725, and 1025 MPa in the non‐deformed, pre‐constant‐loaded, and pre‐fatigued specimens, respectively. The pre‐fatigued specimen tested at the original *s*_max‐W_ was further repeated with the repolish/fatigue test procedure, incrementally increasing *s*_max_ by 25–100 MPa every 10^7^ cycles until reaching 1250 MPa (see methods and Figure S2, Supporting Information): the pre‐fatigued + coaxing specimen with an extraordinary high *s*_max‐W_ of 1300 MPa. The term “coaxing” is used in reference to the “coaxing effect,” where a specimen fatigued at *σ*_W_ does not fracture even after additional fatigue tests at slightly higher stress levels. However, the degree and mechanism of the present coaxing is completely different from the conventional one as discussed in the following sections. The *σ*_B_ of the non‐deformed, pre‐constant‐loaded, and pre‐fatigued specimens were ≈1.6 GPa (Figure S3, Supporting Information). Though the pre‐fatigued + coaxing specimen showed slight softening, the softening was minimal, maintaining a *σ*_B_ ≥ 1.5 GPa. The *s*_max‐W_ was converted to *σ*_W_ at *R* = 0 (*σ*_W(0)_) using the modified‐Goodman diagram^[^ ^29^ ^]^ and plotted in Figure 1a. As‐quenched martensitic steels typically exhibit *σ*_W_ = ≈0.27*σ*_B_ or lower^[^ ^24^, ^25^, ^30^, ^31^ ^]^ (open black circle), notably lower than tempered martensitic steels. The non‐deformed specimen (solid black circle) showed a similar trend, with *σ*_W_ = 0.20*σ*_B_. The *σ*_W_ significantly improved in the pre‐fatigued (blue triangle, *σ*_W_ = 0.31*σ*_B_) and pre‐fatigued + coaxing (red triangle, *σ*_W_ = 0.43*σ*_B_) specimens, successfully overcoming the fatigue limit ceiling. However, improvement was minimal in the pre‐constant‐loaded specimen (green square, *σ*_W_ = 0.22*σ*_B_). The results suggest that fatigue deformation, conventionally considered harmful, can enhance fatigue resistance.

### 2.2 New Coaxing Effect on Crack Initiation

In carbon steels, the coaxing effect has been attributed to dynamic strain aging of carbon,^[^ ^32^, ^33^ ^]^ which hardens the non‐propagation crack tip and enhances crack‐termination ability. Conventionally, the coaxing effect is effective only with small stress increments (below ≈5 MPa). In contrast, the stress increments in this study were exceptionally large (up to 100 MPa), suggesting a new mechanism. Figure S4 (Supporting Information) shows montages of ≈1700 optical microscopy images of the entire gauge part of the non‐deformed specimen tested at the *s*_max‐W_. The dark contrasts are contamination (enlarged in Figure S4e, Supporting Information), and no surface crack was observed. Similarly, no surface cracks appeared in any non‐fractured pre‐deformed specimens tested at their *s*_max‐W_. Therefore, we concluded that the fatigue limit of the as‐quenched martensitic steels corresponded to the crack initiation limit, not the non‐propagation limit. This suggests that pre‐fatigue deformation and subsequent repolishing induced a novel coaxing effect on crack initiation. Understanding this novel coaxing effect would establish new design concepts for superior fatigue crack initiation resistance.

### 2.3 Mechanism of Surface Crack Initiation

**Figure 2:**
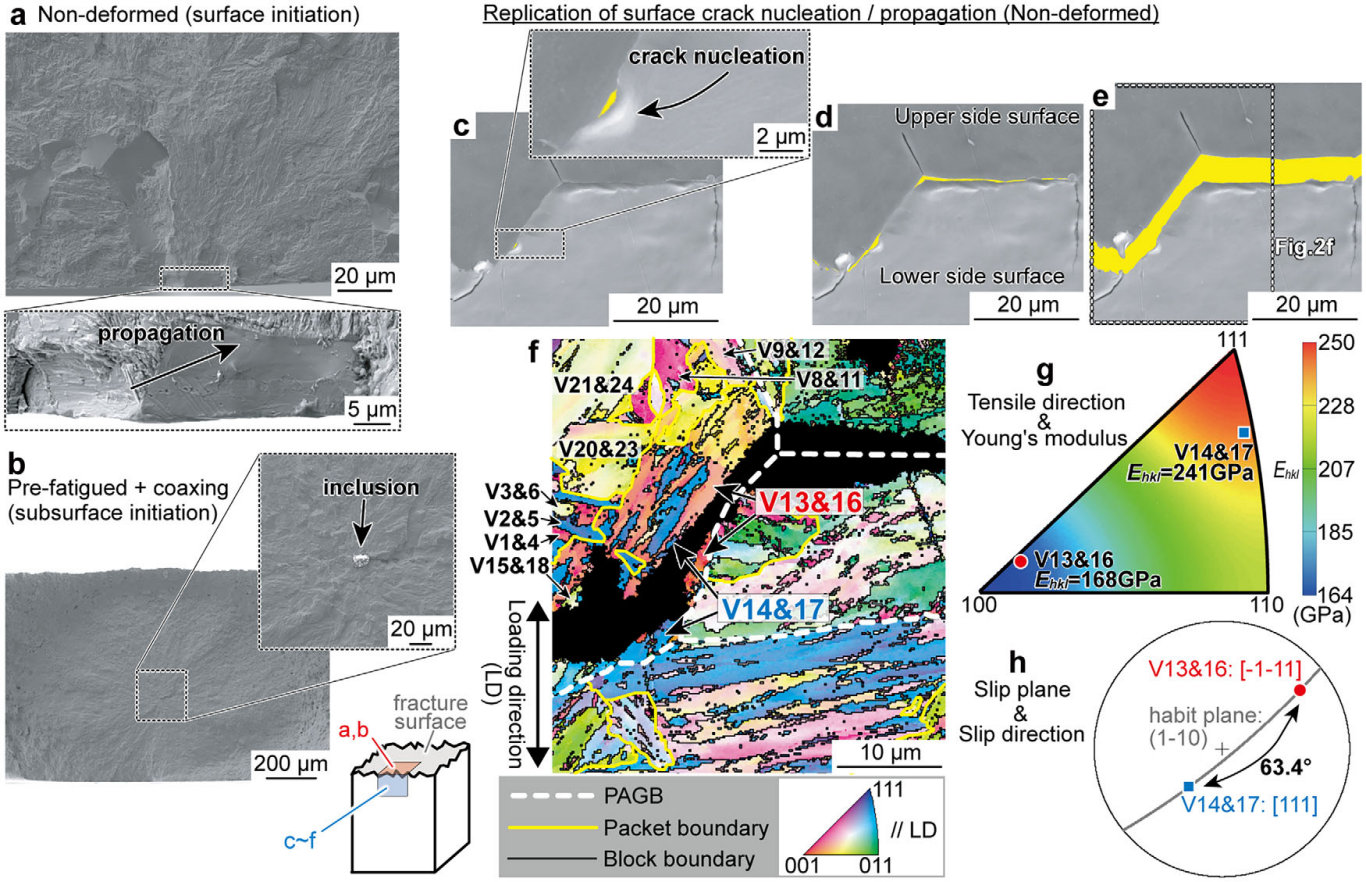
Initiation site of the main fatigue crack in the high‐strength martensitic steel. SEM images of the fracture surface around the a) surface crack initiation site in the non‐deformed specimen tested at *s*_max_ = 700 MPa and b) subsurface crack initiation site in the pre‐fatigued + coaxing specimen tested at *s*_max_ = 1325 MPa. c–e) Replication of surface crack initiation/propagation behavior in the non‐deformed specimen using the surface FRASTA method. SEM images of the upper/lower side surfaces corresponding to the enlarged fracture surface in (a) were used. The cracked region is highlighted in yellow. f) EBSD orientation map corresponding to the white broken rectangle in (e), where the color of each martensite variant (V) represents the crystallographic orientation along the loading direction (LD). Prior austenite grain boundaries (PAGBs), packet boundaries, and block boundaries are indicated by white broken, yellow, and black lines, respectively. g) Stereographic triangle showing Young's modulus in each tensile orientation (*E*_hkl_). h) Stereographic projection showing the in‐lath‐plane primary slip systems in V13&16 and V14&17.

**Figure** **2**a shows an SEM image of the fracture surface of the non‐deformed specimen tested at *s*_max_ = 700 MPa (just above *s*_max‐W_). Radial patterns spread out from the enlarged rectangular area. The enlarged view reveals striated patterns on the smooth surface propagating from the lower left to the upper right, indicating crack initiation at the lower left surface. In contrast, the crack initiated at a subsurface inclusion in the pre‐fatigued + coaxing specimen (Figure 2b). Since the fatigue limit of the present martensitic steels corresponded to the crack initiation limit, the results suggest that suppressing surface crack initiation significantly improved the fatigue limit. Namely, the crack initiation limit at the surface was improved and surpassed that at the subsurface. We emphasize that repolishing after the pre‐deformation is equivalent to cutting out smaller specimens from a pre‐deformed material (see Experimental Section), which differs from merely retarding crack initiation. In materials with coarse grains (∼ tens of micrometers), cracks initiate from intrusions/extrusions formed along slip bands within a grain. Removing these intrusions/extrusions by slight polishing forces the material to undergo the same process of re‐forming intrusions/extrusions before crack initiation, thereby extending fatigue life.^[^ ^34^ ^]^ However, the crack initiation mechanism remains unchanged; the slip bands within a grain re‐develop into intrusions/extrusions and further cracks. No improvement in the fatigue limit was reported in the previous study. Namely, the previous report is the mere retardation, not the suppression, of crack initiation. On the other hand, this study is the first to report that pre‐fatigue training and subsequent repolishing fundamentally suppresses and changes the crack initiation mechanism, leading to the significant improvement in the fatigue limit.

To identify the exact crack initiation site in the non‐deformed specimen, the surface crack initiation/propagation was replicated using the surface FRASTA method (see Experimental Section) (Figure 2c–e). SEM images of the upper/lower side surfaces corresponding to the enlarged fracture surface in Figure 2a were used. The cracked region is yellow‐highlighted. The crack initiation site, indicated by a black arrow in Figure 2c, matches the location identified in Figure 2a. Figure 2f is the EBSD orientation map corresponding to the white broken rectangle in Figure 2e, where the color of each martensite variant (V) represents the crystallographic orientation along the loading direction (LD). The fatigue crack was initiated within the mixed region of V13&16 (red) and V14&17 (blue) in the upper left prior austenite grain (PAG), propagating parallel to their block boundaries. This result aligns with our previous report that fatigue cracks preferentially initiate at high‐angle GBs in as‐quenched martensitic steel.^[^ ^25^ ^]^ Figure 2g is a stereographic triangle showing Young's modulus in each LD (*E*_hkl_). The *E*_hkl_ in any orientation was calculated from the elastic stiffness coefficients measured by in situ neutron diffraction under tensile loading (see Experimental Section, Figure S5a–c, Supporting Information). The maximum and minimum *E*_hkl_ are 250 GPa (*E*_111_) and 164 GPa (*E*_100_), respectively. The *E*_hkl_ of V13&16 was 168 GPa, much smaller than that of V14&17 (241 GPa), demonstrating a significant elastic misfit. Slip deformation, governed by the stress concentration due to elastic anisotropy rather than the Schmid factor (*S_f_*), has shown by elastoplastic self‐consistent modeling^[^ ^35^ ^]^ and 3D crystal plasticity finite element analysis.^[^ ^36^ ^]^ When two dissimilar materials are bonded, an abnormally high‐stress field (a singular stress field) arises at their interfaces, especially at intersections with free surface.^[^ ^37^ ^]^ A significant difference in *E*_hkl_ means that the neighboring variants are elastically “dissimilar” in the LD, generating a large singular stress field at the GBs, especially at their intersection with the specimen surface. Therefore, we presume that the singular stress field due to the elastic misfit between adjacent variants caused local yielding even at the *s*_max_ (700 MPa), well below the *σ*_0.2_ (1118 MPa). In martensitic steel, the slip system parallel to the lath (habit) plane (in‐lath‐plane slip system) is preferentially activated at small strain regimes (below 8%).^[^ ^38^, ^39^ ^]^ For the {011}<111> slip systems, the primary in‐lath‐plane systems in V13&16 and V14&17 were (1‐10)[‐1‐11] (*S_f_* = 0.444) and (1‐10)[111] (*S_f_* = 0.388), respectively (Figure 2h). The 63.4° angle between these slip directions demonstrates significant plastic incompatibility. At the surface, due to the lower geometrical constraint from adjacent variants, each variant is expected to deform into a shape strongly influenced by the activity of the primary in‐lath‐plane slip system. Therefore, greater plastic incompatibility leads to more pronounced surface roughness—i.e., intrusions/extrusions—at high‐angle GBs, which gradually accelerates crack initiation autocatalytically via additional stress concentration originating from the geometrical roughness.^[^ ^25^ ^]^ Similarly, we confirmed that the main cracks were initiated along the surface high‐angle GBs with significant elastic misfit and plastic incompatibility in the pre‐constant‐loaded (Figure S6, Supporting Information) and pre‐fatigued (Figure S7, Supporting Information) specimens tested just above their *s*_max‐W_. In the pre‐fatigued specimens at *s*_max_ = 1025 MPa, one outlier (short‐life ≈7 × 10^6^ cycles, blue triangle with extended corners in Figure 1b) was confirmed. This would be attributed to the crack initiation at a surface inclusion (Figure S8, Supporting Information). Otherwise, the crack initiation sites were surface high‐angle GBs in all the pre‐fatigued, pre‐constant‐loaded, and non‐deformed specimens. Therefore, in the pre‐fatigued specimen, the increase in the crack initiation limit at surface high‐angle GBs improved the fatigue limit; however, the increase was insufficient to change the crack initiation mechanism. On the other hand, in the pre‐fatigued + coaxing specimen, the crack initiation limit at surface high‐angle GBs increased sufficiently to surpass that at subsurface inclusions, changing in the crack initiation mechanism.

**Figure 3:**
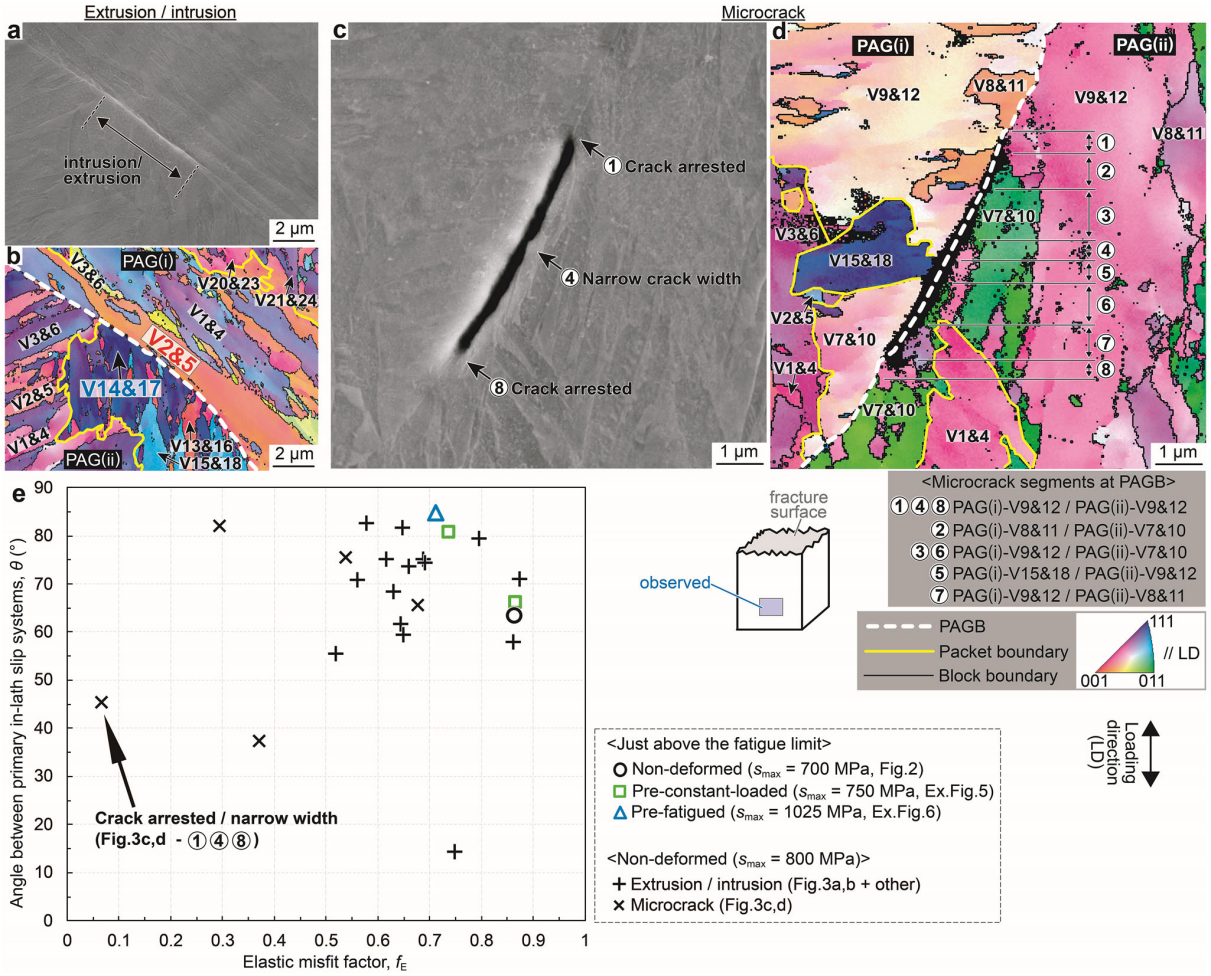
Elastic misfits and plastic incompatibility at the high‐angle grain boundaries where intrusion/extrusion and microcrack were formed. a,c) SEM images and b,d) corresponding EBSD orientation maps of the side surface more than 2 mm away from the fracture surface in the non‐deformed specimen tested at *s*_max_ = 800 MPa: (a, b) and (c, d) show an intrusion/extrusion and a microcrack, respectively. e) Elastic misfit factors and angles between the primary in‐lath‐plane slip systems at the high‐angle boundaries where main cracks (open marks), intrusions/extrusions (plus marks), and microcracks (cross marks) were initiated.

The elastic misfit and plastic incompatibility across intrusion/extrusion (**Figure** **3**a,b) and microcrack (Figure 3c,d) were investigated in the fractured non‐deformed specimen (*s*_max_ = 800 MPa). To discuss the relationship between the fatigue limit and crack initiation mechanisms, specimens tested at stress levels close to the *s*_max‐W_ must be analyzed. Unfortunately, since the *s*_max‐W_ of the non‐deformed specimen is 675 MPa and no microcracks or intrusions/extrusions other than the main crack were observed at stress levels below 800 MPa, all features in the specimens tested at 800 MPa were analyzed. To minimize the stress concentration effects due to main crack propagation, observations were made over 2 mm away from the fracture surface. To quantify elastic misfit, we introduced the elastic misfit factor (*f_E_*), ranging from 0 (no misfit) to 1 (maximum misfit), defined as follows:

(1) $$f_{E}=\frac{\left|E_{\alpha }-E_{\beta }\right|}{E_{111}-E_{100}}$$

where *E*_α_ and *E*_β_ are *E*_hkl_ of variants adjacent to the intrusion/extrusion or crack. Plastic incompatibility was quantified by the angle between primary in‐lath‐plane slip directions (*θ_S_*). In Figure 3a,b, the intrusion/extrusion, indicated by a black arrow, was formed along the PAGB between V2&5 in PAG(i) and V14&17 in PAG(ii), with *f_E_* = 0.87 and *θ_S_* = 70.1°. Similarly, the *f_E_* and *θ_S_* of 14 other intrusions/extrusions were analyzed and plotted in Figure 3e (plus marks). The microcrack along the PAGB (Figure 3c,d) included 8 variant boundary segments (5 variant combinations), whose *f_E_* and *θ_S_*, along with those of the main crack initiation sites (Figure 2, and S7, Supporting Information), are plotted in Figure 3e. Most data points are concentrated in the large *f_E_* and *θ_S_* region, statistically supporting the crack initiation mechanism significantly affected by the elastic misfit and plastic incompatibility. In contrast, segments 1 and 8 arrested the microcrack, and the crack width was exceptionally narrow at segment 4 (Figure 3c), suggesting that these segments retarded crack initiation/propagation. These segments are the PAGB between V9&12 in PAG(i) and V9&12 in PAG(ii), with exceptionally small *f_E_* (0.07). This further highlights the significance of elastic misfit.

Therefore, the singular stress field due to the elastic misfit between the adjacent variants induces local yielding, and, especially at the surface, plastic incompatibility causes intrusion/extrusion (crack embryo) formation. Additional stress concentration originating from the geometrical roughness of intrusions/extrusions accelerates crack initiation. Namely, the precursory sites of crack embryos are the high‐angle GBs with large elastic misfits in LD and plastic incompatibility.

### 2.4 Mechanism of Crack Initiation Suppression

The increase in *s*_max‐W_ does not align with the trends in tensile properties, such as *σ*_B_, proof stresses, and elastic limit, indicating they are not responsible for the crack initiation suppression (Figure S3, Supporting Information). The residual stress relative to the non‐deformed specimen (*σ*_res_), obtained by neutron diffraction (see Experimental Section and Figure S5, Supporting Information), decreases as *s*_max‐W_ increases. However, the reductions (50–100 MPa) are too small to account for the significant increase in *s*_max‐W_ (up to 625 MPa). The results demonstrate that crack initiation, locally occurring in the weakest regions, cannot be explained by macroscopic average properties.

**Figure 4:**
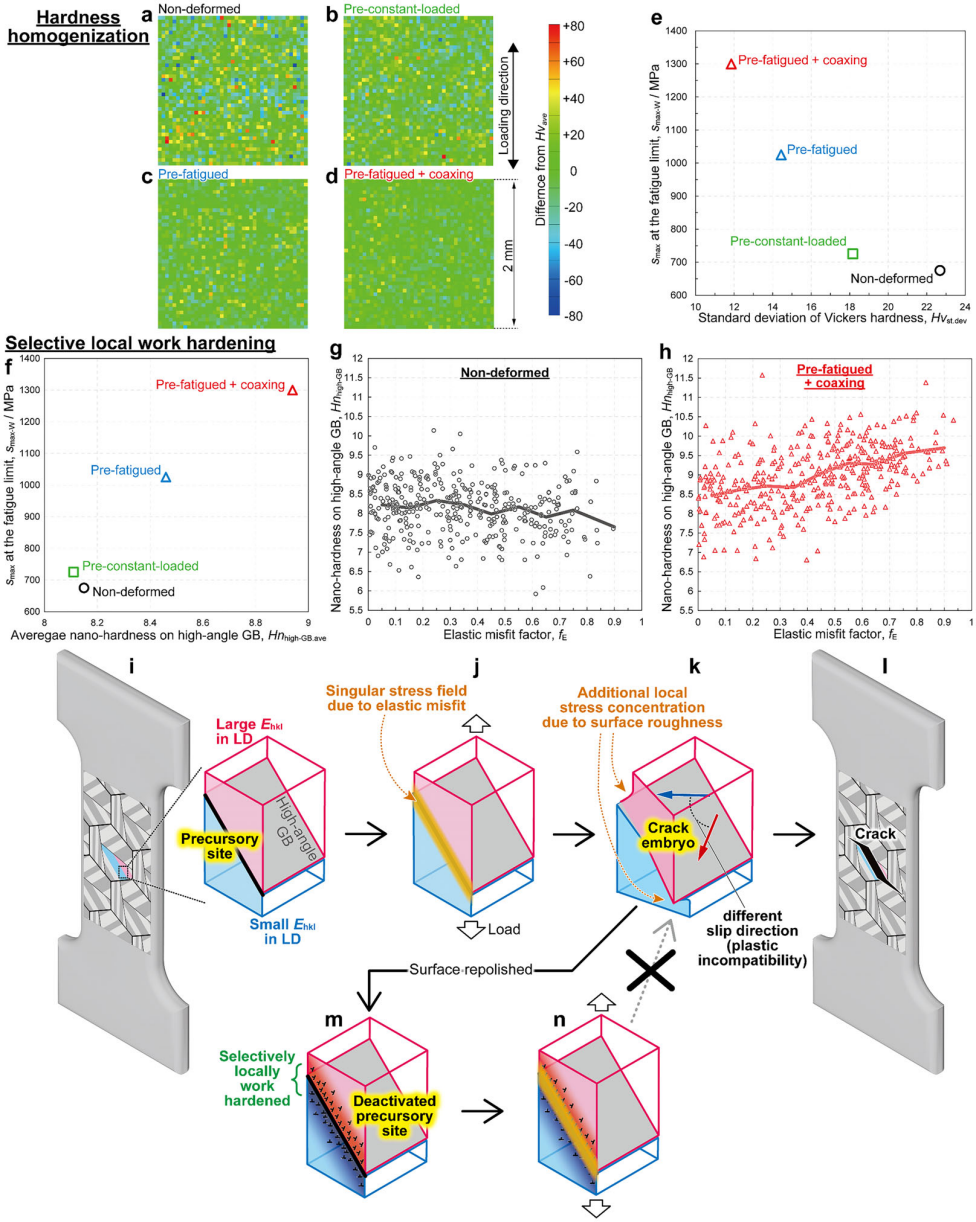
Microstructural self‐optimizations: hardness homogenization and selective local work hardening of precursory site. a–d) Vickers hardness distribution maps where 1681 data points are colored based on the difference from their average value. Relationship between the *s*_max‐W_ and e) the standard deviation of Vickers hardness distribution (*Hv*_st.dev_) or f) the average nano‐hardness on high‐angle GB (*Hn*_high‐GB_). Relationship between the elastic misfit factor and the *Hn*_high‐GB_ in the g) non‐deformed and h) pre‐fatigued + coaxing specimens; all data points (open marks) are plotted with the average values (solid lines). i–n) Schematic illustrations showing the mechanism of the new coaxing effect on crack initiation; i,j) At high‐angle GBs where *E*_hkl_ of the adjacent variants have a large gap (i.e., precursory sites), elastic misfit originates the singular stress field. k) This causes local plastic deformation, forming intrusion/extrusion (i.e., crack embryos). l) Additional stress concentration due to surface roughness leads to crack initiation in the non‐deformed specimen. m,n) However, in the pre‐fatigued (+coaxing) specimens, the crack embryos were removed by repolishing before they became cracks. The precursory sites were deactivated and no longer can become crack embryos because they were selectively work hardened, improving local plastic deformation resistance.

The macroscopic hardness distribution across microstructure and the nano‐hardness corresponding to lath martensitic structure were examined through 1681 micro‐Vickers and 1200 nanoindentation tests, respectively (see Experimental Section and Figure S9, Supporting Information). The average Vickers hardness (*Hv*_ave_) varied little between the specimens and showed no correlation with *s*_max‐W_ (Figure S10a,b, Supporting Information). However, hardness distribution was homogenized by pre‐deformations, visualized in **Figure** **4**a–d with all data points colored by its difference from *Hv*_ave_. The *s*_max‐W_ increased with decreasing the standard deviation of hardness distribution (Figure 4e). Microstructures with wide‐range hardness distributions, such as martensitic‐ferritic dual‐phase steel, lead to the corresponding stress/strain concentrations; softer ferrite undergoes larger plastic deformation, while harder martensite bears higher stress.^[^ ^40^, ^41^ ^]^ Naturally, in the present martensitic steel, the softer areas undergo larger plastic deformation and larger work hardening than the harder areas. Additionally, the reduction in standard deviation without a change in the average value indicates not only the hardening of soft areas but also the softening of hard areas. Therefore, it is possible that the plastic deformation of the softer area released internal residual stress in the harder areas, contributing to hardness homogenization. The local deformation behavior of martensite is affected by the internal residual stress introduced during the martensitic transformation; the release of internal residual stress leads to local softening.^[^ ^42^, ^43^ ^]^ Therefore, one can easily imagine that the hardness homogenization suppressed macro/mesoscale concentration of effective stress/strain (including internal residual and external), suppressing crack initiation.

**Figure 5:**
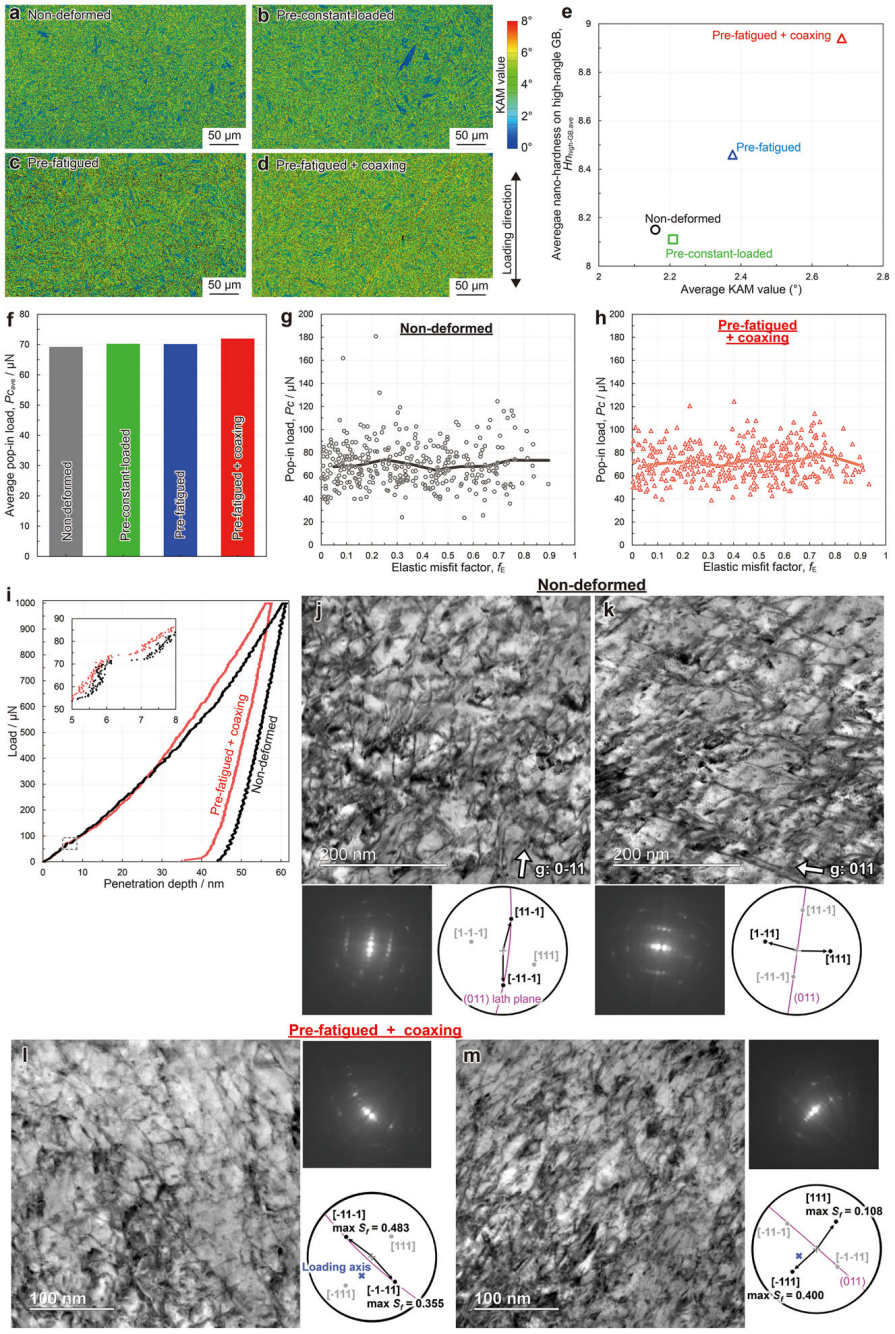
Mechanism of selective nano‐hardening of the precursory sites. a–d) KAM maps of the nanoindentation‐tested regions. e) Relationship between the average KAM value and average *Hn*_high‐GB_. f) Average pop‐in load (*Pc*) on high‐angle GBs in each specimen. Relationship between the elastic misfit factor and the *Pc* on high‐angle GBs in the g) non‐deformed and h) pre‐fatigued + coaxing specimens; all data points (open marks) are plotted with the average values (solid lines). i) Typical load–penetration depth curves on the high‐angle GBs in the non‐deformed (black) and pre‐fatigued + coaxing (red) specimens obtained by the nanoindentation tests. The load–penetration depth curves around the pop‐in (gray broken rectangle) are enlarged. The *Hn*_high‐GB_ was 8.12 and 8.97 in the non‐deformed and pre‐fatigued + coaxing specimens, respectively, which are consistent with the average *Hn*_high‐GB_ in each specimen. j–m) STEM images within laths neighboring to the high‐angle GBs tested in (i): (j, k) non‐deformed and (l, m) pre‐fatigued + coaxing. The observation regions are located ≈5 µm below the nanoindentation measurement surface. (i, k) and (j, l) were observed using *g*‐vectors where only dislocations with Burgers vectors parallel or not parallel to the laths were visible, respectively; Kikuchi diffraction patterns are shown below or right‐side of each image. All the dislocations having a/2<111> Burgers vectors are visible in one of the STEM images. The visible Burgers vectors are indicated in black arrows in each stereographic projection.

All nano‐hardnesses properties were classified as “within lath”, “on lath boundaries”, or “on high‐angle GBs” (see Experimental Section and Figure S10c–f, Supporting Information). The standard deviations of nano‐hardness were minimally affected by pre‐deformations. However, *s*_max‐W_ increased linearly with the average nano‐hardness on high‐angle GBs (*Hn*_high‐GB_) (Figure 4f). To correlate the increase in *Hn*_high‐GB_ with the crack initiation mechanism, all *Hn*_high‐GB_ are plotted against *f_E_* for the non‐deformed (Figure 4g, 355 points) and pre‐fatigued + coaxing (Figure 4h, 377 points) specimens, with their average values indicated by solid lines. The increases in the average and minimum *Hn*_high‐GB_ were more pronounced at high‐angle GBs with larger *f_E_*. **Figure** **5**a–d shows the kernel average misorientation (KAM) maps of the nanoindentation‐tested regions obtained by EBSD. KAM value represents the average misorientation around each measurement point relative to its nearest neighbors. Figure 5e shows the correlation between the average KAM value and average *Hn*_high‐GB_. As the orientation change increases, the average *Hn*_high‐GB_ also increases. Notably, despite the same maximum load during pre‐deformations, the pre‐fatigued specimen exhibits greater orientation changes than the pre‐constant‐loaded specimen. This demonstrates that cyclic loading promoted local plastic deformation, suggesting local work hardening as the origin of the nano‐hardening. The nano‐hardening is unlikely due to the release of internal residual stress. The release of internal residual stress, introduced by the martensitic transformation, leads to a decrease in nano‐hardness.^[^ ^43^ ^]^ The redistribution of carbon, originating from dynamic strain aging, is also unlikely to be the cause of the nano‐hardening. Since carbon atoms are supersaturated in the as‐quenched martensite matrix, the redistribution of carbon is expected to form stable states rather than a further increase in supersaturation:, e.g., dislocation pinning or carbide formation. Dislocation pinning by carbon atoms should increase the critical load for activating dislocation motion (pop‐in load obtained from nanoindentation tests, *Pc*). However, the average *Pc* on high‐angle GBs was largely unaffected by the pre‐deformations (Figure 5f) and the *f_E_* (Figure 5g,h). The representative load–penetration depth curves on high‐angle GBs obtained by the nanoindentation tests are shown in Figure 5i, with the *Hn*_high‐GB_ of 8.12 (black, non‐deformed) and 8.97 (red, pre‐fatigued + coaxing), consistent with each specimen's average. While the *Pc* remains similar, work‐hardening behavior differs. Carbide formation would reduce the matrix hardness, which contradicts the nano‐hardening; additionally, no carbide was observed in the scanning transmission electron microscopy (STEM) images (Figure 5j–m). Figure 5j–m shows STEM images of samples prepared by focused ion beam (FIB) pickup from laths adjacent to the nanoindentation‐tested high‐angle GBs shown in Figure 5i: non‐deformed (j and k) and pre‐fatigued + coaxing (l and m). The observation regions were ≈5 µm below the nanoindentation‐tested surface. Figure 5i–l were observed using *g*‐vectors to visualize dislocations with Burgers vectors either parallel or not parallel to the laths, respectively. No characteristic dislocation structures, such as dislocation cells, were observed in the pre‐fatigued + coaxing specimen. The detailed dislocation‐related mechanism of nano‐hardening could not be fully elucidated. However, we conclude that the high‐angle GBs (and neighboring matrix) with larger *f_E_* undergo greater local plastic deformation to accommodate the singular stress fields, resulting in greater local work hardening.

We propose the mechanism of the new coaxing effect; the precursory sites of crack embryos (surface high‐angle GBs) were selectively plastic‐deformed by a singular stress field, originating from elastic misfit (Figure 4i,j). The local plastic deformation and possibly the resultant release of internal residual stress contributed to the macroscopic hardness homogenization. On the specimen surface, plastic incompatibility led to intrusions/extrusions with considerable magnitude, and at this point, precursory sites became crack embryos — consequently, additional stress concentration by surface roughness led to crack initiation (Figure 4k,l). However, in the pre‐fatigued (+coaxing) specimens, the surface layer including crack embryos was removed by repolishing before they became cracks (Figure 4m). These precursory sites were deactivated and no longer can become crack embryos because they were selectively work hardened in nano‐scale. The macroscopic hardness homogenization mitigated the macro/mesoscale stress/strain concentrations (including both internal residual and external), further contributing to the deactivation. Namely, crack initiation was suppressed by the improved local plastic deformation resistance and the reduction in effective localized stress (Figure 4n).

### 2.5 Crack Embryo Engineering

**Figure 6:**
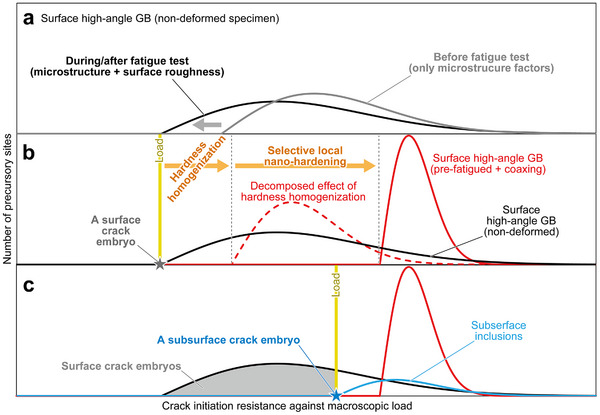
Schematic illustrations showing the crack embryo engineering. a) Schematic illustrations of the distribution of crack initiation resistance against macroscopic load for surface high‐angle GBs in the non‐deformed specimen: before the fatigue test (gray line) and during/after the fatigue test (black line). Schematic illustrations of the resistance distributions at macroscopic stresses corresponding to the fatigue limit of the b) non‐deformed and c) pre‐fatigued + coaxing specimens. The surface high‐angle GBs (precursory sites) corresponding to the resistance distribution lower than the macroscopic load become crack embryos (intrusions/extrusions) and eventually cracks. Crack embryo(s) corresponds to (b) the lowest point of the resistance distribution just above the fatigue limit loading (gray star) and (c) a certain area under a larger loading (gray area). As indicated by red lines, the lowest point of the pre‐fatigued + coaxing specimen was increased by the hardness homogenization and selective local hardening, thereby, deactivating the precursory sites. The red broken line in (b) illustrates the decomposed effect of only the hardness homogenization. Under the fatigue limit loading of the pre‐fatigued + coaxing specimen, macroscopic load corresponds to the lowest point of the resistance distribution for subsurface inclusions (blue line and star).

Weakest‐link theory, a common framework for statistical fracture analysis, posits that the fracture strength of a material is determined by its weakest domains having a certain cracking resistance distribution.^[^ ^44^ ^]^ The domains with a resistance lower than macroscopic load can be cracked. When the fatigue limit corresponds to the crack initiation limit, no domain should have a crack initiation resistance lower than the macroscopic load at the fatigue limit. This study identifies the weakest domains—i.e., precursory sites of fatigue crack embryos—as the surface high‐angle GBs with significant elastic misfit and plastic incompatibility in martensitic steel. **Figure** **6**a illustrates the distribution of crack initiation resistance against macroscopic load for surface high‐angle GBs in the non‐deformed specimen. Before the fatigue test (gray line), all surface high‐angle GBs can, to some degree, be regarded as precursory sites, with resistances varying by microstructural factors such as *f*_E_ and *θ*_S_. Once a load is applied, intrusions/extrusions are, to some extent, formed along the weak precursory sites; the resultant geometrical roughness causes additional stress concentration. Consequently, during/after the fatigue test (black line), the resistance distribution broadens toward smaller macroscopic loads due to this enhanced local stress. Due to the plastic deformation localization, the precursory sites with resistance below the macroscopic load become crack embryos, which autocatalytically become cracks at the same load. Conversely, the precursory sites with resistance above the macroscopic load do not form cracks—even if minor intrusions/extrusions are formed—as elastic misfit and plastic incompatibility (and the resultant roughness‐enhanced stress concentration) are not sufficiently large. Thus, the lowest point of the resistance distribution corresponds to *σ*_W_, leading to nearly single crack initiation just above the *σ*_W_ (Figure 6b, gray star). Under larger loads, many precursory sites can become crack embryos (Figure 6c, gray area), resulting in multiple simultaneous crack initiations. No secondary cracks were found in the non‐deformed specimen tested at *s*_max_ = 700 MPa (just above *s*_max‐W_), while many secondary cracks and intrusions/extrusions appeared at *s*_max_ = 800 MPa (Figure 3). Therefore, the goal of “crack embryo engineering” is to elevate the lowest point of the resistance distribution. Namely, a fatigue‐crack‐initiation‐resistant microstructure minimizes localized plastic deformation at specific sites—either by reducing the effective local stress or enhancing the local plastic deformation resistance of precursory sites—both of which directly influence the resistance distribution.

Controlling the intrinsic *f*_E_ and *θ*_S_ of a material could greatly influence the resistance distribution but requires crystallography control by novel alloy design, thermomechanical treatments, or both. In contrast, the present study used pre‐fatigue deformations for controlling the resistance distribution and demonstrated two effective crack embryo engineering strategies applicable to general high‐strength steels. First, homogenizing macroscopic hardness distribution (Figure 4a–e) narrowed the resistance distribution, elevating its lowest point without changing the mean value (Figure 6b, red broken line). Since the average *Hv* remained constant, the average resistance did not improve. However, the hardness homogenization resulted from plastic deformation (and work hardening) of relatively soft regions during pre‐deformation, and possibly from the release of internal residual stress, reducing variations in intrinsic plastic deformation resistance and effective stress/strain concentration (including internal residual and external) in the macro/mesoscale. Second, the plastic deformation resistance of the weak precursory sites was selectively increased by the nano‐hardening (Figure 4f–n), directly elevating the lowest point. The combination of these effects significantly elevated the lowest point and deactivated the weak precursory sites (Figure 6b, red solid line), thereby suppressing the plastic deformation localization at these sites—that is, the cyclic‐training‐driven self‐optimization. These self‐optimization effects should intrinsically occur in the non‐deformed specimens during fatigue test, while being offset by the crack embryos (i.e., surface roughness and resultant stress concentration). The amplitude of intrusions/extrusions (crack embryos) formed in an initial few cycles significantly impacts the fatigue limit.^[^ ^45^ ^]^ This study successfully isolated the self‐optimization effects by removing crack embryos. Consequently, the lowest point for surface high‐angle GBs exceeded that for subsurface inclusions (Figure 6c, blue line), changing the crack initiation mechanism (Figure 2a,b). The detailed mechanism of fatigue crack initiation at subsurface inclusions remains unclear. However, since this study did not adopt an approach to remove subsurface crack embryos, the crack initiation limit at subsurface inclusions would not be improved by the pre‐deformations. Thus, controlling the lowest point of the resistance distribution is crucial for improving crack initiation resistance, not necessarily required to change the macroscopic average properties, such as tensile strength, ductility, etc. It is essential to deeply understand and suppress the formation mechanism of crack embryos among the crack initiation process of “precursory site → embryo → crack”; this is the essence of crack embryo engineering. Revealing “what constitutes a crack embryo” in the crack initiation at subsurface inclusion (precursory site) would further allow for controlling the subsurface crack initiation.

## 3 Conclusion

This study proposes a novel strategy, crack embryo engineering, to improve crack initiation resistance, demonstrating a breakthrough in overcoming the conventional fatigue limit ceiling in high‐strength as‐quenched martensitic steel; the maximum stress corresponding to the fatigue limit significantly increased from 675 to 1300 MPa by pre‐fatigue training with minimal change in tensile strength. In the as‐heat‐treated state, high‐angle boundaries with large elastic misfits and plastic incompatibility served as precursory sites for intrusions/extrusions (crack embryos), eventually leading to crack initiation. However, after the pre‐fatigue training and subsequent repolishing, surface crack initiation was entirely suppressed by the intrinsic microstructural self‐optimization of (pre‐)fatigue deformation: macroscopic hardness homogenization and selective nano‐hardening of the precursory sites. The self‐optimization deactivated the precursory sites, i.e., the formation of intrusion/extrusion was suppressed, effectively controlling the lowest point of the resistance distribution for crack initiation. Crack embryo engineering is a novel material design framework that subdivides the crack initiation process into “precursory site → embryo → crack” and aims to suppress crack initiation by systematically understanding and controlling the stages before crack embryo formation. The present microstructural self‐optimization against fatigue deformation is a successful crack embryo engineering strategy, doubling the fatigue limit with minimal change in the macroscopic average properties. This demonstrates that fatigue deformation, conventionally considered harmful, can be a beneficial and versatile approach to enhancing fracture resistance in general steels, providing an alternative to tempering heat treatment that inevitably sacrifices tensile strength.

## 4 Experimental Section

### Material

A Fe‐3Mn‐0.2C ingot (Mn: 3.02, C: 0.18, Si: 0.01, Al: 0.002, S: 0.001, P: <0.002, N: 0.002, O: 0.001, and Fe: balance (wt.%)) was used in the present study. The hot‐rolled plate was cold‐rolled from 18 to 1.8 mm in thickness (reduction: 90%). The cold‐rolled sheet was austenitized at 900 °C for 30 min, ice brine quenched, and then sub‐zero cooled in liquid nitrogen for 10 min.

### Fatigue and Tensile Tests

Figure S2a (Supporting Information) shows the pre‐deformation procedures. Sheet‐type smooth specimens with a gauge length of 8 mm, width of 4 mm, and thickness of 1 mm were fabricated from the as‐heat‐treated specimen (Figure S2b, Supporting Information). The specimens were mechanically wet‐polished using emery paper from #80 to #4000 and finished by electrochemical polishing in an aqueous solution of 450 ml CH_3_COOH + 50 ml HCLO_4_ at 22 V for 90 s (referred to as non‐deformed specimen). The non‐deformed specimens were uniaxially fatigue‐deformed at maximum stress (*s*_max_) of 1000 MPa, frequency of 50 Hz, and stress amplitude of 50 MPa (referred to as pre‐fatigued specimen) or that of 0 MPa (referred to as pre‐constant‐loaded specimen) for 10^7^ cycles (2 × 10^5^ s). A 20–30 µm surface layer was removed by mechanical and electrochemical repolishing, followed by a fatigue test at a frequency of 50 Hz and stress ratio (*R*) of 0.1. It is important to emphasize that, given that a martensite lath (a single crystal) is submicron in size, repolishing the 20–30 µm surface layer is equivalent to cutting out new small specimens from a larger pre‐deformed material. As a result, the surface‐specific plastic deformation history (including geometrical roughness) during pre‐deformation was removed, and all grains from the surface to the subsurface in the repolished specimen were originally inside the material during the pre‐deformations, having experienced (macroscopically) uniform training. This is fundamentally distinct from methods like shot peening, which induces a residual stress gradient from the surface to the interior. Due to the limited load capacity of the testing machine, repolishing was performed for each specimen. As shown in Figure 1b, the *s*_max_ corresponding to the fatigue limit (*s*_max‐W_) of the non‐deformed, pre‐constant‐loaded, pre‐fatigued specimens were 675, 725, and 1025 MPa, respectively. For the pre‐fatigued specimens tested at their *s*_max‐W_, the repolish/fatigue test procedure with an increase in *s*_max_ by 25–100 MPa was repeated every 10^7^ cycles until they fractured. The pre‐fatigued specimens finally fractured at *s*_max_ ≥ 1325 MPa. The pre‐fatigued specimen tested up to *s*_max_ = 1250 MPa was used as the initial microstructure of the pre‐fatigued + coaxing specimen. Consequently, the *s*_max‐W_ was 1300 MPa in the pre‐fatigued + coaxing specimen. For comparison, the *σ*_W_ of the present martensitic steels at *R* = 0.1 was converted to those at *R* = 0 using the modified Goodman diagram,^[^ ^29^ ^]^ plotted in Figure 1a.

(2) $$\sigma_{W(x)}=\sigma_{W(-1)}\left(1-\frac{\sigma_{m(x)}}{\sigma_{B}}\right)$$

where *σ*_B_ is tensile strength, *σ*_m(x)_ and *σ*_W(x)_ are the average stress and stress amplitude, respectively, corresponding to the fatigue limit at *R* = x. The fatigue test at the fatigue limit was repeated twice to confirm no fracture below that load. Uniaxial tensile tests were also performed at an initial strain rate of 10^−4^ s^−1^ to evaluate tensile strength, proof stresses, and elastic limit. The elastic limit was defined as the nominal stress where the nominal stress–nominal strain curve fell by more than 5 MPa below a straight line extrapolated from the slope between 50 and 250 MPa.

It should be noted that an *R*‐value of 0.1 was selected to preserve the fracture surface morphology. At *R* ≤ 0, upper/lower fracture surfaces may come into contact and deform during unloading or compression, which could significantly reduce the accuracy of the fracture surface topography analysis (FRASTA) method explained later. It was also noted that the sheet‐type specimen, rather than the round‐bar specimen, was intentionally used because the flat surface facilitate the analysis of surface crack initiation behavior using optical microscopy, scanning electron microscopy (SEM, ZEISS: sigma), and electron backscattered diffraction (EBSD). While edge stress concentration in the sheet‐type specimen may lower the *σ*_W_, it does not lead to an overestimation of *σ*_W_, ensuring the reliability of the excellent *σ*_W_ of the pre‐fatigued + coaxing specimen shown in Figure 1.

### Surface FRASTA Method

FRASTA is a methodological approach that computationally reconstructs microscopic fracture processes by analyzing the topographic characteristics of corresponding areas on opposing fracture surfaces.^[^ ^46^, ^47^ ^]^ Typically, FRASTA utilizes reconstructed 3D geometry data of the fracture surfaces. However, when the sample surface serves as the crack initiation site, identifiable from the fracture surface, 3D data was unnecessary. This study introduced the “surface FRASTA method” specifically for analyzing surface crack initiation. SEM images of upper/lower surfaces around the crack initiation site were used. These images were superimposed until aligned with no visible gap, after which the relative distance between them was incrementally increased, resulting in the appearance of gaps between the two conjugate surface images. The appeared gaps likely correspond to fractured regions, enabling the replication of surface crack initiation/propagation by sequentially increasing the relative distance. The surface crack initiations/propagations, shown in Figure 2c–e, Figures S6b–d, S7b–d, and S8c–e (Supporting Information), were replicated using the surface FRASTA method. The fractured regions were yellow‐highlighted. The crystallographic orientations of the corresponding areas were measured using EBSD. The EBSD measurement (step size: 0.1 µm, acceleration voltage: 15 kV) and analysis were performed with the Bruker QUANTAX‐EBSD system and the TSL OIM Analysis program, respectively. Additionally, for the specimens that did not fracture after the fatigue test, the absence of surface cracks was confirmed using optical microscopy (OLYMPUS: DSX1000). The entire gauge part was examined by stitching together ≈1700 images (each ≈240 µm square) of the front, back, left, and right surfaces (Figure S4, Supporting Information).

### Lath Martensitic Structure and Variant Analysis

The lath martensite structure is a characteristic microstructure in high‐strength low/medium‐carbon steels. It is well known that lath martensite satisfies the Kurdjumov‐Sachs (K‐S) orientation relationship relative to parent austenite, given by {111}_A_//{011}_M_ and <101>_A_//<111>_M_, where the subscripts A and M denote austenite and martensite, respectively. In the K‐S orientation relationship, twenty‐four equivalent crystallographic variants can be transformed from a single austenite grain.^[^ ^48^, ^49^ ^]^ This diversity of crystallographic variants leads to the subdivision of an austenite grain into several structural units with different size scales, namely lath, sub‐block, block, and packet.^[^ ^50^, ^51^ ^]^ A martensite lath is a single crystal of martensite ≈0.2 µm thick, with misorientation between adjacent laths being less than 5°. A sub‐block is an aggregation of laths of an identical variant. A block consists of a specific combination of two variants with a small misorientation, such as V1‐V4, V2‐V5, or V3‐V6. The boundaries between adjacent laths (habit planes) or adjacent blocks correspond to crystallographic planes approximately parallel to {011}, satisfying the parallel plane relationship in the K‐S orientation relationship. A packet comprises six variants (V1 to V6, V7 to V12, V13 to V18, or V19 to V24) that exhibit the same parallel plane relationship in the K‐S orientation relationship. Typically, several packets are present within each prior austenite grain (PAG). Due to the distinct crystallographic orientations of the martensite variants, crystallographic analysis can precisely characterize the lath martensite structure. In this study, block boundaries, packet boundaries, and PAG boundaries (PAGB) were classified as high‐angle boundaries. It did not differentiate between sub‐block and lath boundaries, as sub‐block boundaries were not clearly distinguishable after deformation. The average PAG size of the present martensitic steel measured by the line‐interception method was 62 µm.

### In Situ Neutron Diffraction Experiment During Tensile Loading

In situ neutron diffraction experiments were conducted during tensile loading to measure elastic constants and residual stress of the martensitic steels. These experiments were performed at the Materials and Life Science Experimental Facility (MLF) of the Japan Proton Accelerator Research Complex (J‐PARC), using the Engineering Materials Diffractometer, TAKUMI. At this facility, it was possible to measure both the lattice spacing (*d*) in the loading direction (LD), which expands under tensile stress, and that in the transverse direction (TD), which shrinks under tensile loading. Detailed information about the TAKUMI can be found in the literature.^[^ ^52^ ^]^ A uniaxial tensile test was conducted on the non‐deformed specimen at an initial strain rate of 1.3 × 10^−5^ s^−1^. The tensile load was held constant for 5 min at various stress levels from 0 to 550 MPa. The specimen surface was randomly sprayed with a black pattern, which was captured every 5 s to accurately calculate the nominal strain in the LD using the digital image correlation (DIC) method. The diffraction profiles in the LD (Figure S5a, Supporting Information) and TD (Figure S5b, Supporting Information) for each holding stage are shown with true stress indicated on the right side. The lattice strains of each lattice plane (*ε*_hkl_) were calculated by the following equation:

(3) $$\varepsilon_{hkl}=\frac{d_{hkl}-d_{0-hkl}}{d_{0-hkl}}$$

where *d*_0‐hkl_ is the *d* at 0 MPa. The *d* of each lattice plane was determined by fitting the diffraction profiles to a Voigt function. The *ε*_hkl_ as a function of true stress in the LD and TD are shown in Figure S5c (Supporting Information). Young's modulus (*E*_hkl_) and Poisson's ratio (*ν*_hkl_) of each lattice plane were determined from least‐squares fit slopes, as summarized in Table S1 (Supporting Information). The bulk Young's modulus (*E*_bulk_) and Poisson's ratio (*ν*_bulk_) were calculated to be 213.6 GPa and 0.293, respectively, using the following equations:

(4) $$E_{bulk}=\sum_{hkl}I_{LD-hkl}E_{hkl}$$

(5) $$\nu_{bulk}=\sum_{hkl}I_{TD-hkl}\nu_{hkl}$$

where *I*_LD‐hkl_ and *I*_TD‐hkl_ are the normalized integrated intensities of each *hkl* profile in the LD and TD, respectively. Below 550 MPa, plastic deformation was minimal, so the *I*_LD‐hkl_ and *I*_TD‐hkl_ remained unchanged. Elastic stiffness coefficients (*s*_11_, *s*_12_, and *s*_44_) were determined from the following simultaneous equations:^[^ ^53^ ^]^

(6) $$\frac{1}{E_{hkl}}=s_{11}-a\frac{h^{2}k^{2}+k^{2}l^{2}+l^{2}h^{2}}{\left(h^{2}+k^{2}+l^{2}\right)}$$

(7) $$a=2s_{11}-2s_{12}-s_{44}$$

(8) $$\frac{1-2\nu_{bulk}}{E_{bulk}}=s_{11}+2s_{12}$$

The Equation (6) was solved by the least‐squares approximation using all *E*_hkl_ in Table S1 (Supporting Information). The obtained values were *s*_11_ = 6.074, *s*_12_ = ‐2.065, and *s*_44_ = 10.063 TPa^−1^. Finally, *E*_hkl_ for any arbitrary LD was calculated using the following equations^[^ ^54^ ^]^ and is shown in Figure 2G, Figures S6f, and S7f (Supporting Information).

(9) $$E_{hkl}=\frac{1}{s_{11}+\left(2s_{12}-2s_{11}+s_{44}\right)H^{2}}$$

(10) $$H^{2}=\frac{h^{2}k^{2}+k^{2}l^{2}+l^{2}h^{2}}{\left(h^{2}+k^{2}+l^{2}\right)}$$

Neutron diffraction experiments were also performed on the pre‐deformed specimens in the unloaded state. As illustrated by the 110 peaks in Figure S5d (Supporting Information), the *d*_0_ values of the pre‐deformed specimens were clearly reduced. The relative residual stresses of each lattice plane (*σ*_res‐hkl_) and bulk average (*σ*_res‐bulk_) relative to the non‐deformed specimen were calculated using the following equations:

(11)

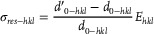

(12) $$\sigma_{res-bulk}=\sum_{hkl}I_{LD-hkl}\sigma_{res-hkl}$$

where *d’*_0‐hkl_ is the lattice spacing in the pre‐deformed specimens in the unloaded state. The residual stresses are presented in Figure S5e (Supporting Information). The *σ*_res_ value is 0 in the non‐deformed specimen and is not shown in Figure S5e (Supporting Information) because it is the reference value.

### Multiscale Hardness Tests

The macroscopic distribution of hardness across microstructure and the nano‐hardness corresponding to lath martensitic structure were examined by conducting the micro‐Vickers and nanoindentation tests, respectively. The micro‐Vickers tests were performed with a maximum load of 490.3 mN and a holding period of 10 s, using the SHIMADZU HMV‐G test machine. The resultant indentation size was ≈13 µm, which was larger than the average block width of ≈3 µm. A total of 1681 hardness measurements were taken for each specimen, with tests conducted every 50 µm within a 2 mm square area. Optical microscopy images of the entire measurement area and an enlarged view of it in the non‐deformed specimen are shown in Figure S9a,b (Supporting Information), respectively. For the nanoindentation testing, the Bruker Hysitron Triboindenter TI950, equipped with a Berkovich indenter, was used. The tests were conducted in load‐control mode with a maximum load of 1000 µN and a loading rate of 50 µN s^−1^, resulting in an indentation size of ≈300 nm. A total of 1200 tests were performed for each specimen, with tests conducted every 7 µm within the area (203 × 273 µm) that was measured by EBSD in advance (Figure S9c, Supporting Information). Subsequently, 300 backscattered electron (BSE) images of the corresponding area were captured, each containing four indentations. By comparing the BSE images (Figure S9d, Supporting Information) with the corresponding EBSD orientation maps (Figure S9e, Supporting Information), the position of each indentation was classified into within lath, on lath boundaries, or on high‐angle GBs. It should be noted that the affected areas around the nanoindentations were not considered, and the indentations overlapping with the boundaries were classified as “on the boundary”. Using a focused ion beam (FIB, ZEISS: Crossbeam), thin foils were lifted out from the nanoindentation‐tested surface. The lifted‐out samples were observed by scanning transmission electron microscopy (STEM, JEOL: JEM‐2800) at an acceleration voltage of 200 kV.

## Conflict of Interest

The authors declare no conflict of interest.

## Author Contributions

K.O., K.T., and A.S. performed conceptualization and methodology. K.O. and E.N. performed investigation and data analysis. K.O. and A.S. performed funding acquisition and Project administration. K.T. and A.S. performed supervision. K.O. performed wrote the original draft. K.T., E.N., and A.S. performed wrote, reviewed, edited the draft.

## Supporting information



Supporting Information

## Data Availability

The data that support the findings of this study are available from the corresponding author upon reasonable request.

## References

[1] H. Mughrabi , Philos. Trans. R. Soc. A 2015, 373, 20140132. 10.1098/rsta.2014.0132 25713457

[2] A. Pineau , D. L. McDowell , E. P. Busso , S. D. Antolovich , Acta Mater. 2016, 107, 484.

[3] X. Guo , Y. Dong , J. Qin , Q. Zhang , H. Zhu , S. Zhu , Adv. Mater. 2025, 37, 2312816. 10.1002/adma.202312816 38445902

[4] J. Liu , W. Li , Y. She , S. Blanchard , S. Lin , Adv. Mater. 2024, 10.1002/adma.202407925 PMC12691901 39328076

[5] D. Raabe , C. C. Tasan , E. A. Olivetti , Nature 2019, 575, 64. 31695209 10.1038/s41586-019-1702-5

[6] J. W. Morris Jr. , Nat. Mater. 2017, 16, 787. 28748963 10.1038/nmat4949

[7] a) NIMS Fatigue Data Sheet, 1982.

[8] a) NIMS Fatigue Data Sheet, 1982.

[9] Y. Furuya , H. Nishikawa , H. Hirukawa , N. Nagashima , E. Takeuchi , Sci. Technol. Adv. Mater. 2019, 20, 1055. 31762842 10.1080/14686996.2019.1680574 PMC6853246

[10] M. S. Rashid , Science 1980, 208, 862. 17772810 10.1126/science.208.4446.862

[11] P. Vivegananthan , S. Gao , W. Ji , H. Fan , C. Han , K. Zhou , Adv. Mater. 2024, 36, 2402130. 10.1002/adma.202402130 39420709

[12] S. Fliegener , J. Rosenberger , M. Luke , J. M. Domínguez , J. F. Morgado , H. U. Kobialka , T. Kraft , J. Tlatlik , Adv. Eng. Mater. 2024, 27, 2400992.

[13] R. O. Ritchie , Int. Met. Rev. 1979, 24, 205.

[14] R. Pippan , C. Zelger , E. Gach , C. Bichler , H. Weinhandl , Fatigue Fract. Eng. Mater. Struct. 2011, 34, 1.

[15] M. Koyama , Z. Zhang , M. Wang , D. Ponge , D. Raabe , K. Tsuzaki , H. Noguchi , C. C. Tasan , Science 2017, 355, 1055. 28280201 10.1126/science.aal2766

[16] Y. Murakami , M. Endo , Int. J. Fatigue 1994, 16, 163.

[17] J. C. Newman , E. P. Phillips , M. H. Swain , Int. J. Fatigue 1999, 21, 109.

[18] Z. Qu , Z. Zhang , R. Liu , L. Xu , Y. Zhang , X. Li , Z. Zhao , Q. Duan , S. Wang , S. Li , Y. Ma , X. Shao , R. Yang , J. Eckert , R. O. Ritchie , Z. Zhang , Nature 2024, 626, 999. 38418915 10.1038/s41586-024-07048-1

[19] C. Dan , Y. Cui , Y. Wu , Z. Chen , H. Liu , G. Ji , Y. Xiao , H. Chen , M. Wang , J. Liu , L. Wang , Y. Li , A. Addad , Y. Zhou , S. Ma , Q. Shi , H. Wang , J. Lu , Nat. Mater. 2023, 22, 1182. 37592031 10.1038/s41563-023-01651-9

[20] J. Sun , H. Li , Y. Chen , X. An , Adv. Sci. 2024, 11, 2407283.

[21] P. D. Niu , R. D. Li , K. F. Gan , Z. Q. Fan , T. C. Yuan , C. J. Han , Adv. Mater. 2024, 36, 2310160. 10.1002/adma.202310160 38489830

[22] P. Shi , Y. Li , X. Jiang , Z. Shen , R. Li , Z. Lin , Q. Li , B. Ding , T. Zheng , X. Liang , N. Min , J. Peng , H. Li , W. Ren , Z. Lei , Y. Ren , C. T. Liu , Y. Zhong , E. Ma , Adv. Mater. 2024, 36, 2405459. 10.1002/adma.202405459 38847443

[23] M. A. S. Torres , H. J. C. Voorwald , Int. J. Fatigue 2002, 24, 877.

[24] N. Iwata , Y. Tomota , K. Katahira , H. Suzuki , Mater. Sci. Technol. 2002, 18, 629.

[25] K. Okada , A. Shibata , Y. Takeda , N. Tsuji , Int. J. Fatigue 2021, 143, 105921.

[26] G. Seidametova , J. B. Vogt , I. P. Serre , Int. J. Fatigue 2018, 106, 38.

[27] H. A. Padill II , B. L. Boyce , Exp. Mech. 2010, 50, 5.

[28] S. Suresh , Fatigue of Materials, Cambridge University, Cambridge 1991.

[29] T. Nicholas , J. R. Zuiker , Int. J. Fract. 1996, 80, 219.

[30] H. Matsumiya , A. Shibata , K. Okada , N. Tsuji , Int. J. Hydrogen Energy 2021, 46, 37509.

[31] K. Monma , H. Sudo , J. Jpn. Inst. Met. Mater. 1963, 27, 125.

[32] T. Yakushiji , M. Kage , H. Nisitani , Trans. Jpn. Soc. Mech. Eng. Ser. A 1996, 62, 82.

[33] M. Koyama , B. Ren , N. Yoshimura , E. Sakurada , K. Ushioda , H. Noguchi , ISIJ Int. 2017, 57, 358.

[34] N. Thompson , N. Wadsworth , N. Louat , Philos. Mag. 1956, 1, 113.

[35] B. Clausen , T. Lorentzen , T. Leffers , Acta Mater. 1998, 46, 3087.

[36] C. J. Neil , J. A. Wollmershauser , B. Clausen , C. N. Tomé , S. R. Agnew , Int. J. Plast. 2010, 26, 1772.

[37] S. Ioka , K. Masuda , S. Kubo , Int. J. Solids Struct. 2007, 44, 6232.

[38] S. Nambu , M. Michiuchi , Y. Ishimoto , K. Asakura , J. Inoue , T. Koseki , Scr. Mater. 2009, 60, 221.

[39] M. Michiuchi , S. Nambu , Y. Ishimoto , J. Inoue , T. Koseki , Acta Mater. 2009, 57, 5283.

[40] S. Zarei , R. J. Nedoushan , M. Atapour , Mater. Sci. Eng. A 2016, 674, 384.

[41] M. Park , Y. Fujimura , A. Shibata , N. Tsuji , Mater. Sci. Eng., A 2024, 916, 147301.

[42] B. Hutchinson , D. Lindell , M. Barnett , ISIJ Int. 2015, 55, 1114.

[43] D. Fukui , N. Nakada , S. Onaka , Acta Mater. 2020, 196, 660.

[44] W. Weibull , J. Appl. Mech. 1951, 13, 293.

[45] J. C. Stinville , M. A. Charpagne , A. Cervellon , S. Hemery , F. Wang , P. G. Callahan , V. Valle , T. M. Pollock , Science 2022, 377, 1065. 36048948 10.1126/science.abn0392

[46] T. Kobayashi , D. A. Shockey , Eng. Fract. Mech. 2010, 77, 2370.

[47] A. Shibata , T. Matsuoka , A. Ueno , N. Tsuji , Int. J. Fract. 2017, 205, 73.

[48] S. Morito , X. Huang , T. Furuhara , T. Maki , N. Hansen , Acta Mater. 2006, 54, 5323.

[49] S. Morito , H. Tanaka , R. Konishi , T. Furuhara , T. Maki , Acta Mater. 2003, 51, 1789.

[50] A. R. Marder , G. Krauss , Trans. Am. Soc. Met. 1967, 60, 651.

[51] A. Shibata , G. Miyamoto , S. Morito , A. Nakamura , T. Moronaga , H. Kitano , I. G. Urrutia , T. Hara , K. Tsuzaki , Acta Mater. 2023, 246, 118675.

[52] S. Harjo , T. Ito , K. Aizawa , H. Arima , J. Abe , A. Moriai , T. Iwahashi , T. Kamiyama , Mater. Sci. Forum 2011, 681, 443.

[53] H. Dannoshita , H. Hasegawa , S. Higuchi , H. Matsuda , W. Gong , T. Kawasaki , S. Harjo , O. Umezawa , Mater. Sci. Eng. A 2022, 854, 143795.

[54] R. Ueji , A. Shibata , K. Ushioda , Y. Kimura , T. Ohmura , T. Inoue , Scr. Mater. 2021, 194, 113666.

