# Supplementary material for: Fatigue Limit Doubling in High‐Strength Martensitic Steel through Crack Embryo Engineering–Cyclic‐Training‐Driven Self‐Optimization
Source: Adv Sci (Weinh). 2025 Jun 29;12(33):e04165. doi: 10.1002/advs.202504165 (PMC12412530; doi:10.1002/advs.202504165)
Supplement: Supplementary file 1 — Supporting Information [file ADVS-12-e04165-s001.pdf]

ADVANCED SCIENCE Open Access

Supporting Information

for *Adv. Sci.*, DOI 10.1002/advs.202504165

# Fatigue Limit Doubling in High-Strength Martensitic Steel through Crack Embryo Engineering–Cyclic-Training-Driven Self-Optimization

*Kazuho Okada*, Kaneaki Tsuzaki, Eri Nakagawa and Akinobu Shibata*

# Supporting Information

**Fatigue limit doubling in high-strength martensitic steel**
**through crack embryo engineering–cyclic-training-driven self-optimization**

*Kazuho Okada*, Kaneaki Tsuzaki, Eri, Nakagawa, Akinobu Shibata*

## Upper limit size of non-propagation crack

There is a widely accepted empirical formula for predicting the $\sigma_{W(-1)}$ of the materials containing pre-existing surface defects, proposed by Murakami et al.[16], as follows:

$$\sigma_{W(-1)} = 1.43 \frac{Hv + 120}{\left(\sqrt{area}\right)^{1/6}} \qquad \text{(with ±10\% error)} \tag{13}$$

where *Hv* is the Vickers hardness and *area* is the projected area (μm$^2$) of a surface defect in LD. Using the following empirical formula, *Hv* can be transformed into $\sigma_B$:

$$\sigma_B = 3.12Hv + 16 \tag{14}$$

Combining the above formulas (13 and 14) with the proportional relationship between $\sigma_{W(-1)}$ and $\sigma_B$ ($\sigma_{W(-1)} = 0.53\sigma_B$[9]), we can calculate the upper limit size of the non-propagation surface crack necessary for achieving the proportional relationship at any arbitrary $\sigma_B$, as shown in **Figure S1**. In other words, once a surface crack is initiated, it must be terminated below the upper limit size to maintain the $\sigma_W$–$\sigma_B$ proportional relationship. The errors, indicated by broken lines in **Figure S1**, originate from the ±10% error in the formula (13).

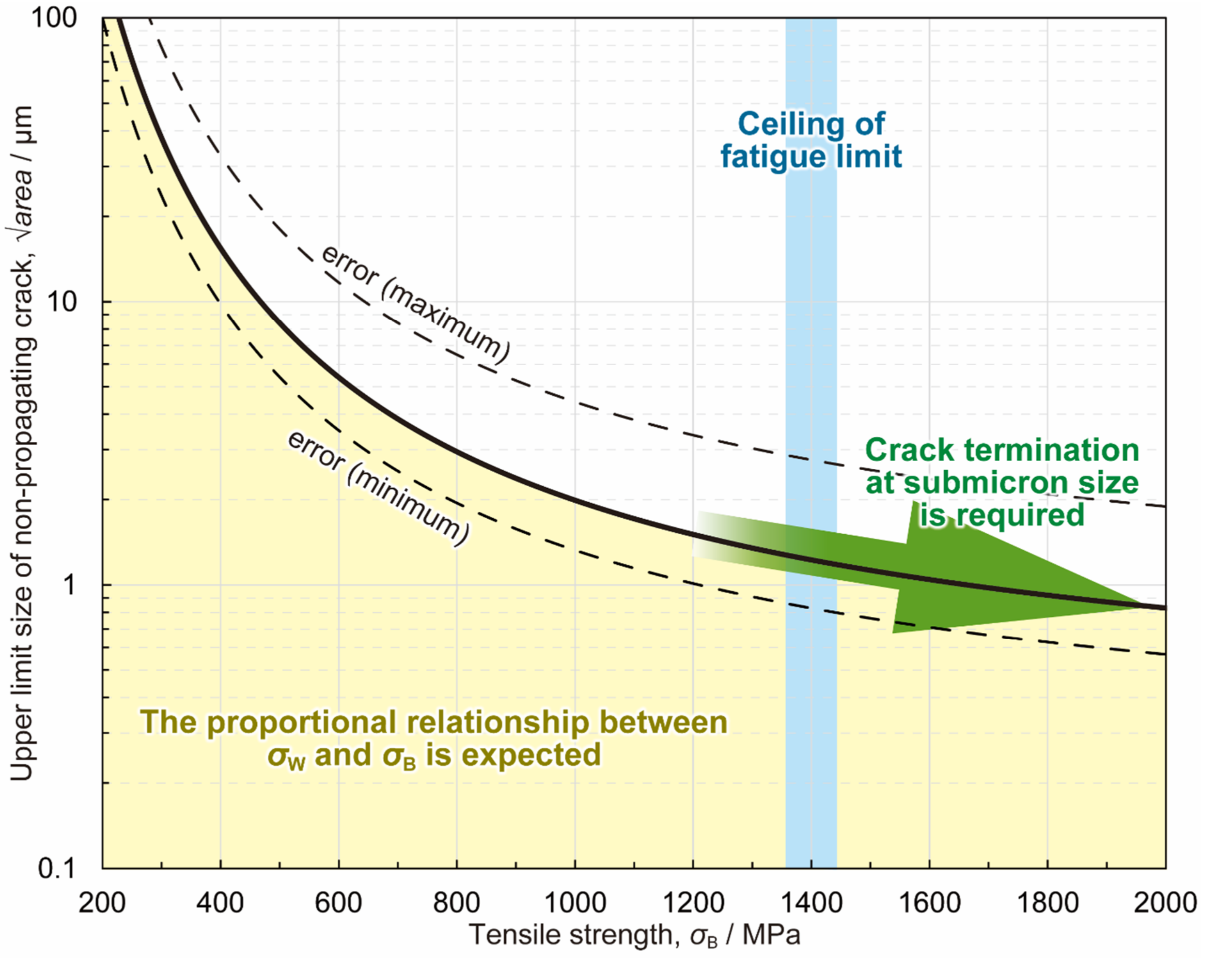


**Figure S1: Ceiling of the crack non-propagation limit.** Relationship between the tensile strength ($\sigma_B$) and the upper limit size of the non-propagation crack (√area) which is necessary for achieving the proportional relationship between tensile strength and fatigue limit at $R$ = -1 ($\sigma_{W(-1)}$).

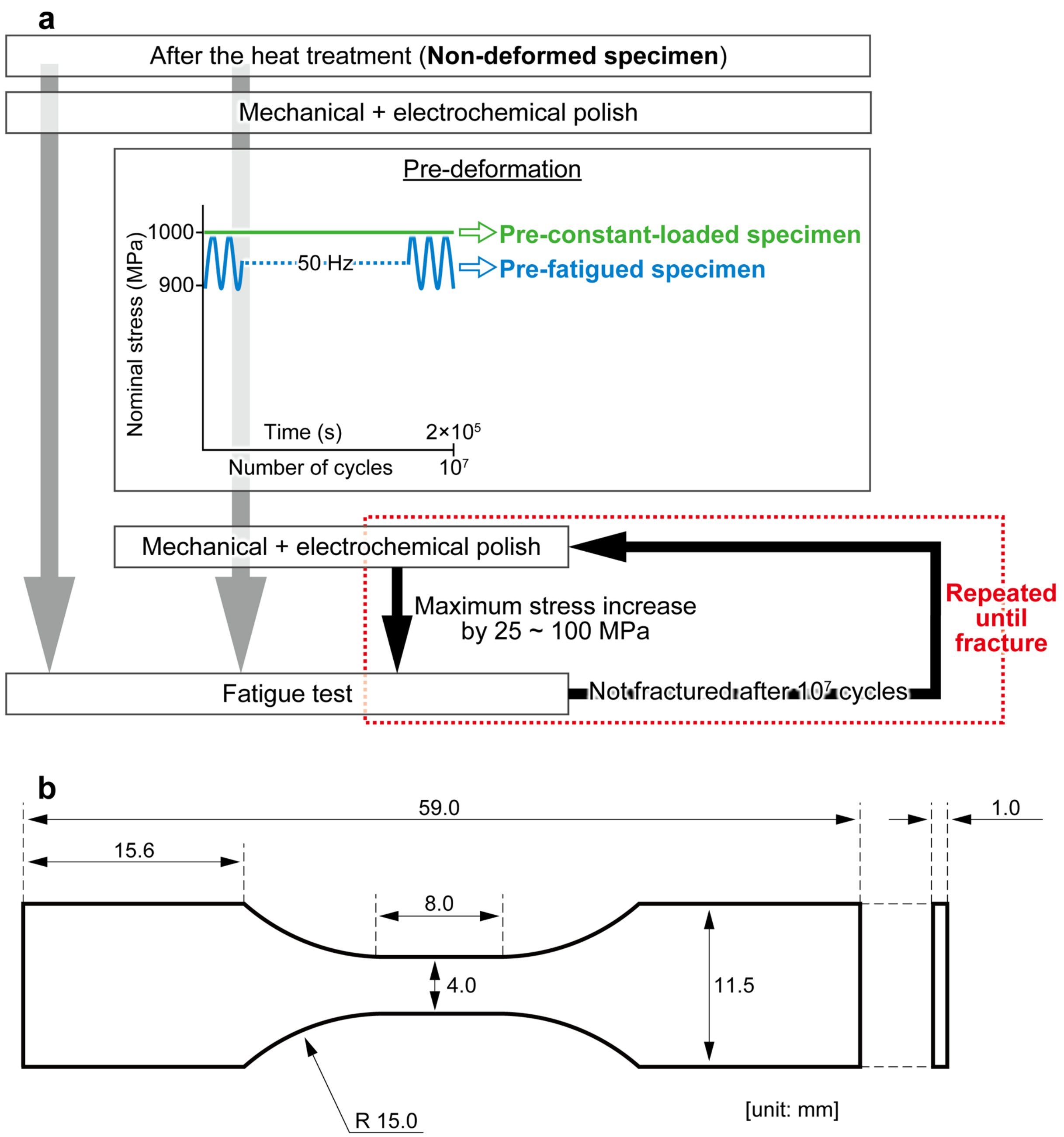


**Figure S2: Details of the (pre-)deformations.** Schematic illustrations of the (a) pre-deformation procedures and (b) sheet-type smooth specimen.

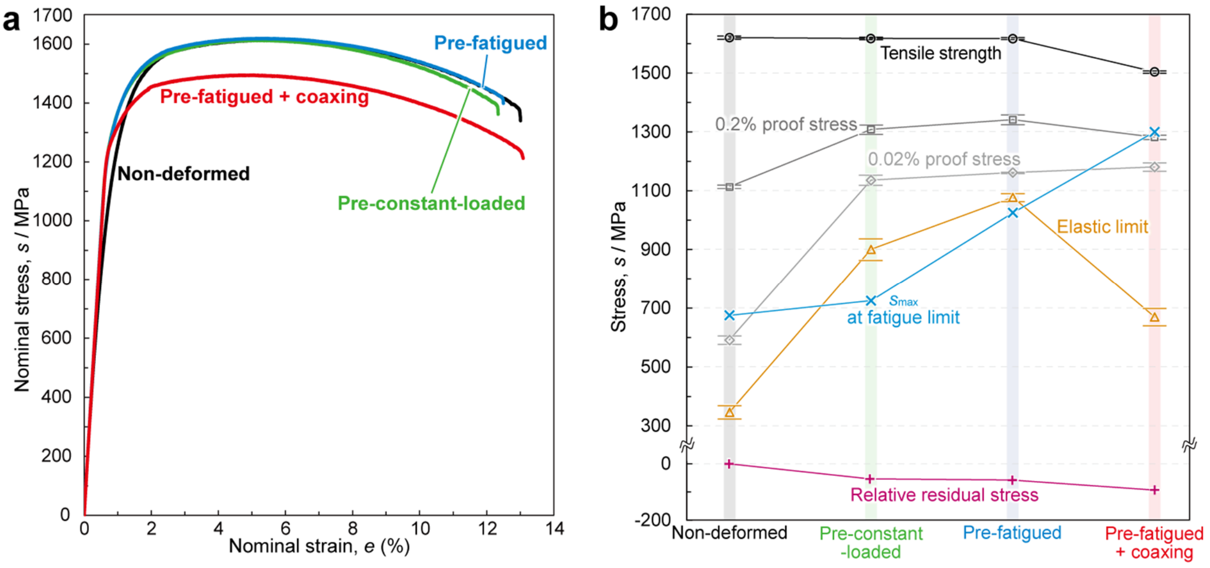


**Figure S3: Relationship between the fatigue limit and macroscopic average mechanical properties.** (a) Nominal stress – nominal strain curves of the non-deformed (black), pre-constant-loaded (green), pre-fatigued (blue), and pre-fatigued + coaxing (red) specimens. (b) Summary of the macroscopic mechanical properties: tensile strength (black circle), 0.2%/0.02% proof stresses (gray square/diamond), elastic limit (orange triangle), relative residual stress against non-deformed specimen (pink plus mark), and $s_{max}$ corresponding to the fatigue limit ($s_{max\text{-}w}$, blue cross mark).

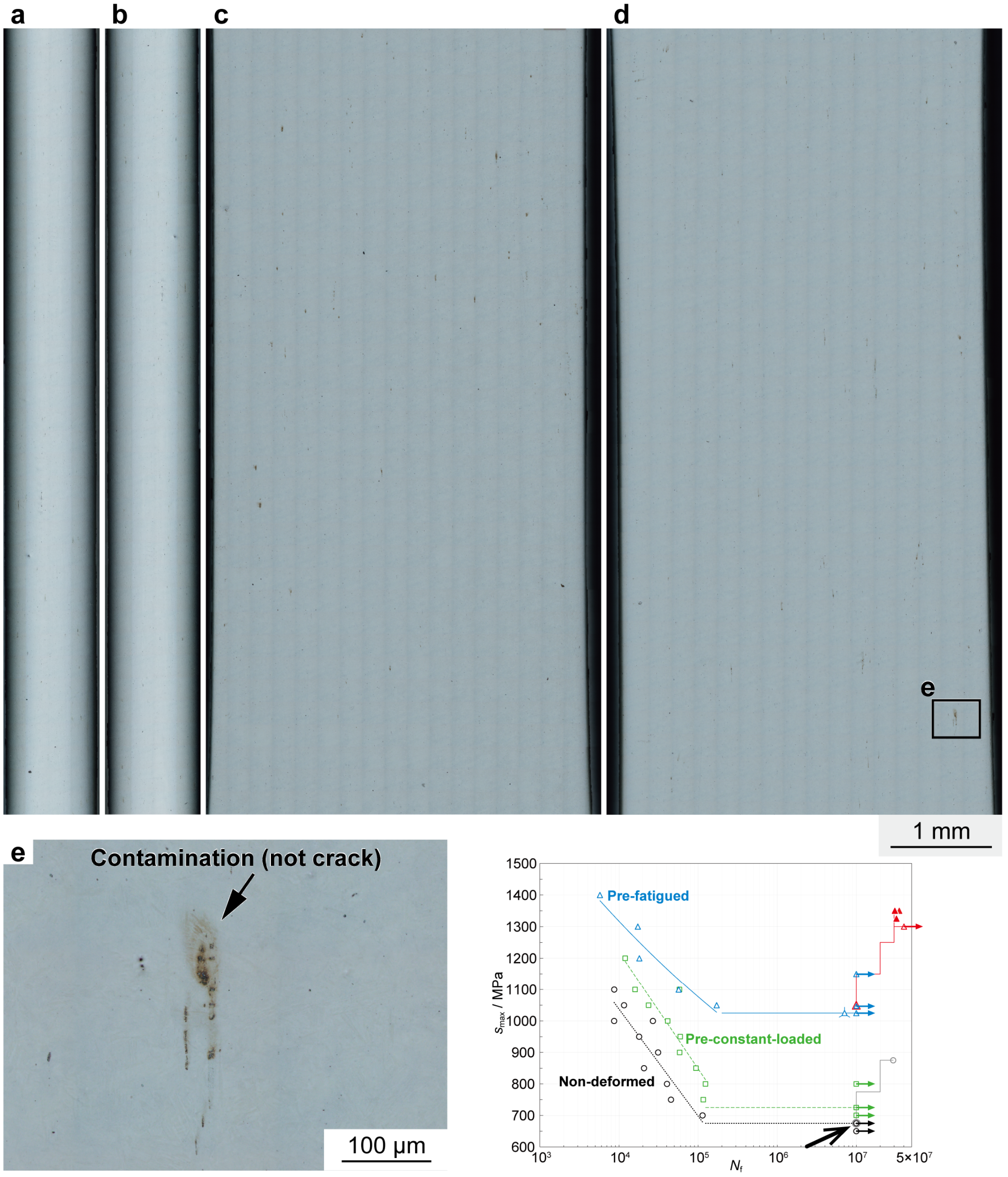


**Figure S4: Absence of surface crack at the fatigue limit.** Optical microscopy images of the entire gauge part in the non-deformed specimen tested at the fatigue limit ($s_{max}$ = 675 MPa, $10^7$ cycles): (a,b) right/left surfaces and (c, d) front/back surfaces. 1700 images (each of them ~240 μm square) in total were stuck together. (e) Enlarged view of the black rectangle in (d); the dark contrast is the oil contamination or oxidization during the fatigue test and no surface crack was observed.

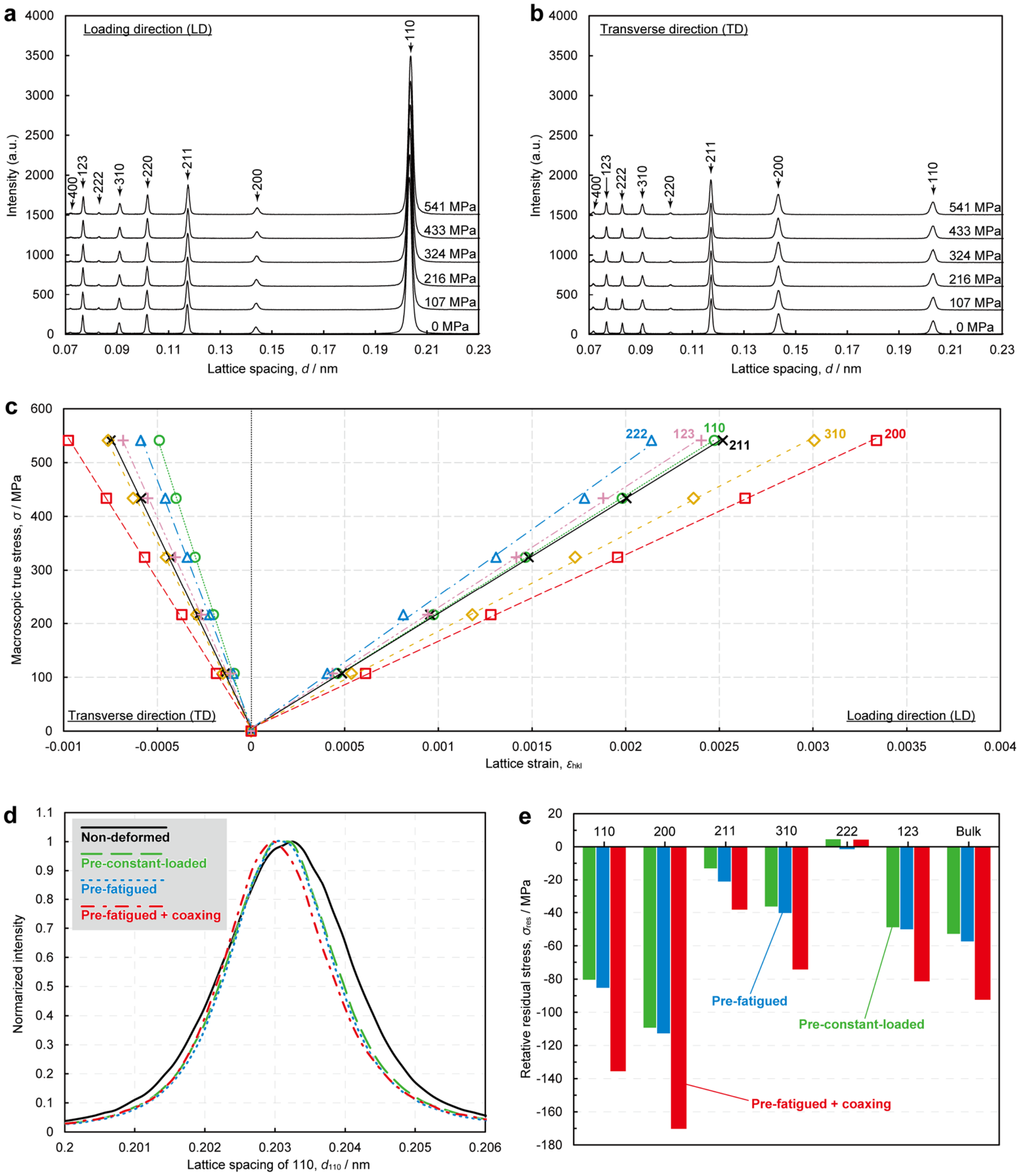


**Figure S5: Measurements of elastic constants and residual stress.** *In situ* neutron diffraction profiles ((a) loading direction (LD) and (b) transverse direction (TD) and (c) lattice strains during tensile loading in the non-deformed specimen. (d) Neutron diffraction profiles of 110 planes at non-loaded state. (e) Relative residual stress in the LD of each lattice plane and bulk average relative to the non-deformed specimen in the pre-deformed specimens.

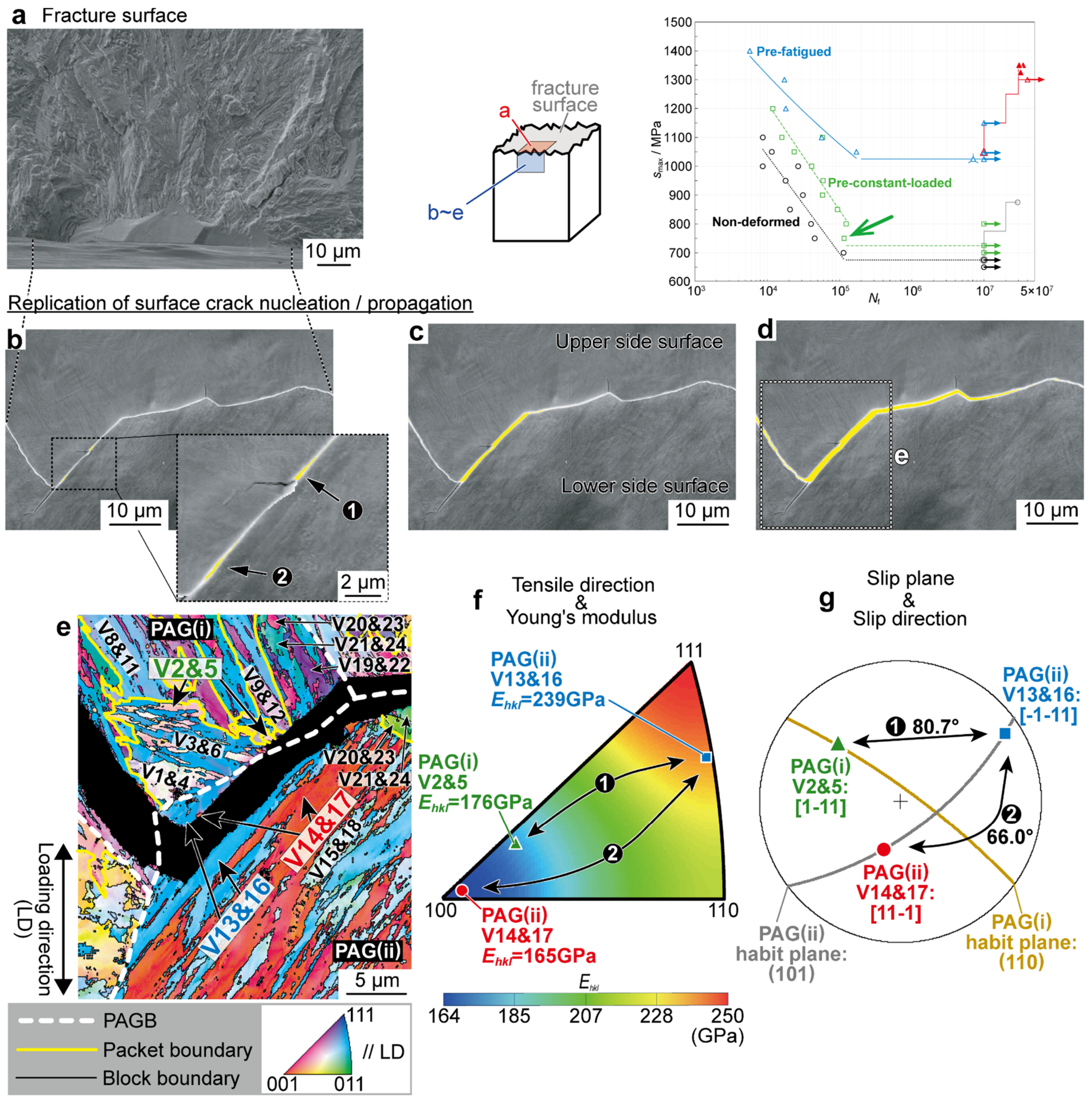


**Figure S6: Initiation site of the main fatigue crack in the pre-constant-loaded specimen.** (a) SEM image of the fracture surface around the surface crack initiation site in the pre-constant-loaded specimen ($s_{max}$ = 750 MPa). (b–d) Replication of surface crack initiation/propagation; the surface crack was simultaneously initiated at sites 1 and 2 indicated by yellow highlights in the enlarged view of (b). (e) EBSD orientation map corresponding to the white broken rectangle in (d). The crack initiation site 1 was the PAGB between V2&5 in PAG(i) and V13&16 in PAG(ii). Another crack was initiated at site 2 approximately parallel to the block boundaries in the mixed region of V13&16 and V14&17 in PAG(ii). (f) Stereographic triangle showing the tensile directions and corresponding Young's modulus ($E_{hkl}$) in the loading direction. Large elastic misfits were confirmed at both crack initiation sites. (g) Stereographic projection showing the in-lath-plane primary slip systems. The angles between the in-lath primary slip directions of the adjacent variants at sites 1 and 2 were 80.7° and 66.0°, respectively.

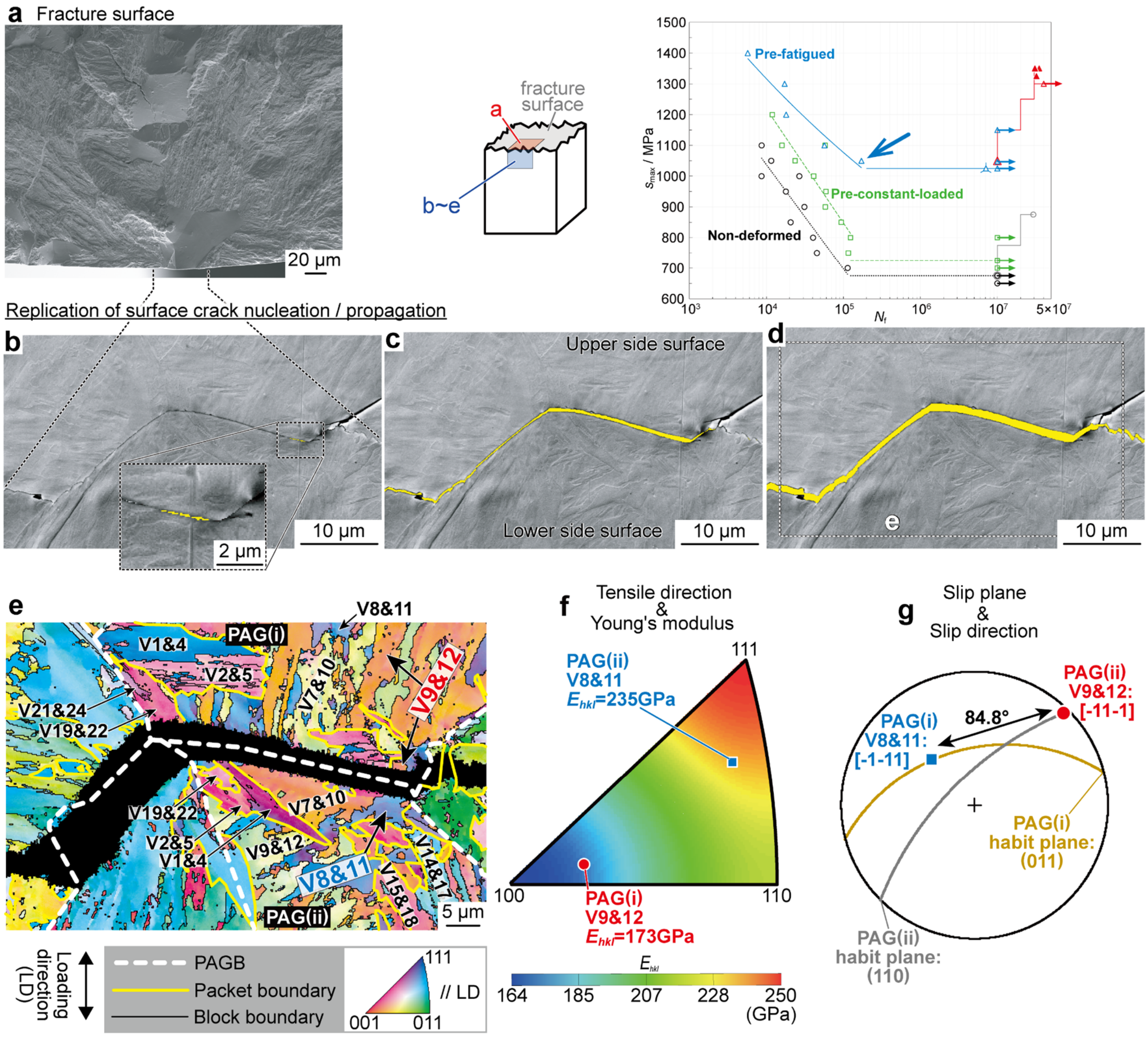


**Figure S7: Initiation site of the main fatigue crack in the pre-fatigued specimen ($s_{max}$ = 1050 MPa).** (a) SEM image of the fracture surface around the surface crack initiation site in the pre-fatigued specimen ($s_{max}$ = 1050 MPa). (b–d) Replication of surface crack initiation/propagation in which a yellow background indicates the cracking. (e) EBSD orientation map corresponding to the white broken rectangle in (d). The surface crack was initiated at PAGB between V9&12 in PAG(i) and V8&11 in PAG(ii). (f) Stereographic triangle showing the tensile directions and corresponding Young's modulus ($E_{hkl}$) in the loading direction. (g) Stereographic projection showing the in-lath-plane primary slip systems. Large elastic/plastic misfits were confirmed at the crack initiation site.

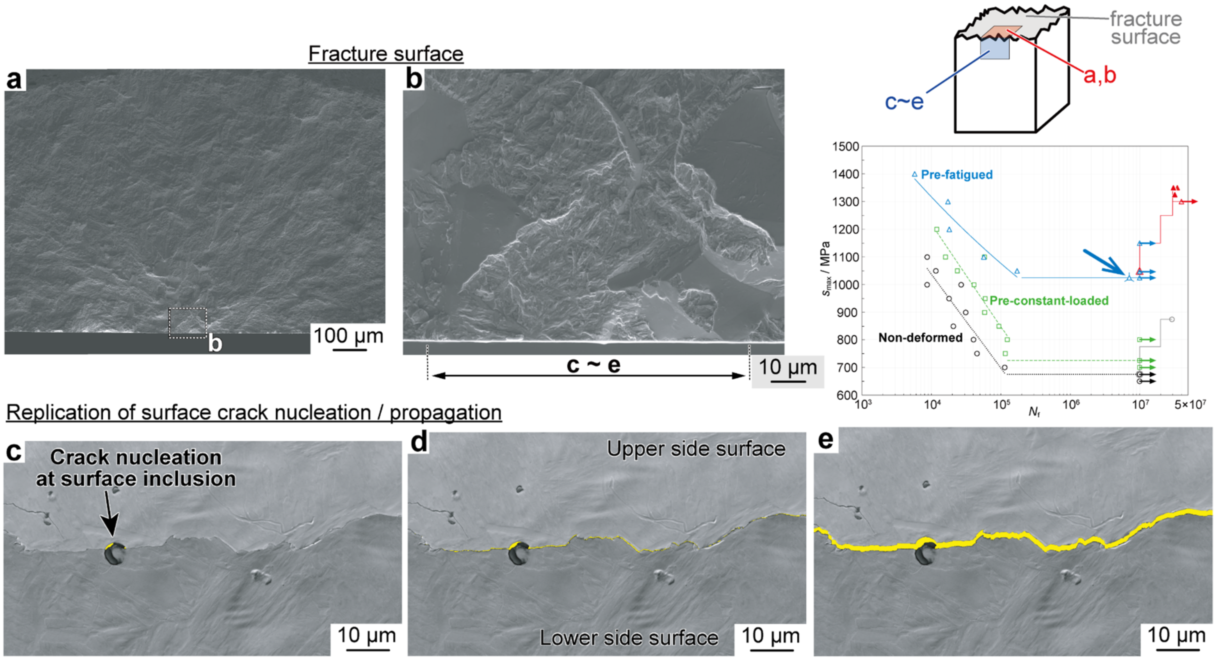


**Figure S8: Initiation site of the main fatigue crack in the pre-fatigued specimen ($s_{max}$ = 1025 MPa).** (a) SEM image of the fracture surface around the surface crack initiation site in the pre-fatigued specimen ($s_{max}$ = 1025 MPa). (b) Enlarged view of the white dotted rectangle in (a). (c–e) Replication of surface crack initiation/propagation in which a yellow background indicates the cracking. The surface crack was initiated at the interface between the matrix and inclusion.

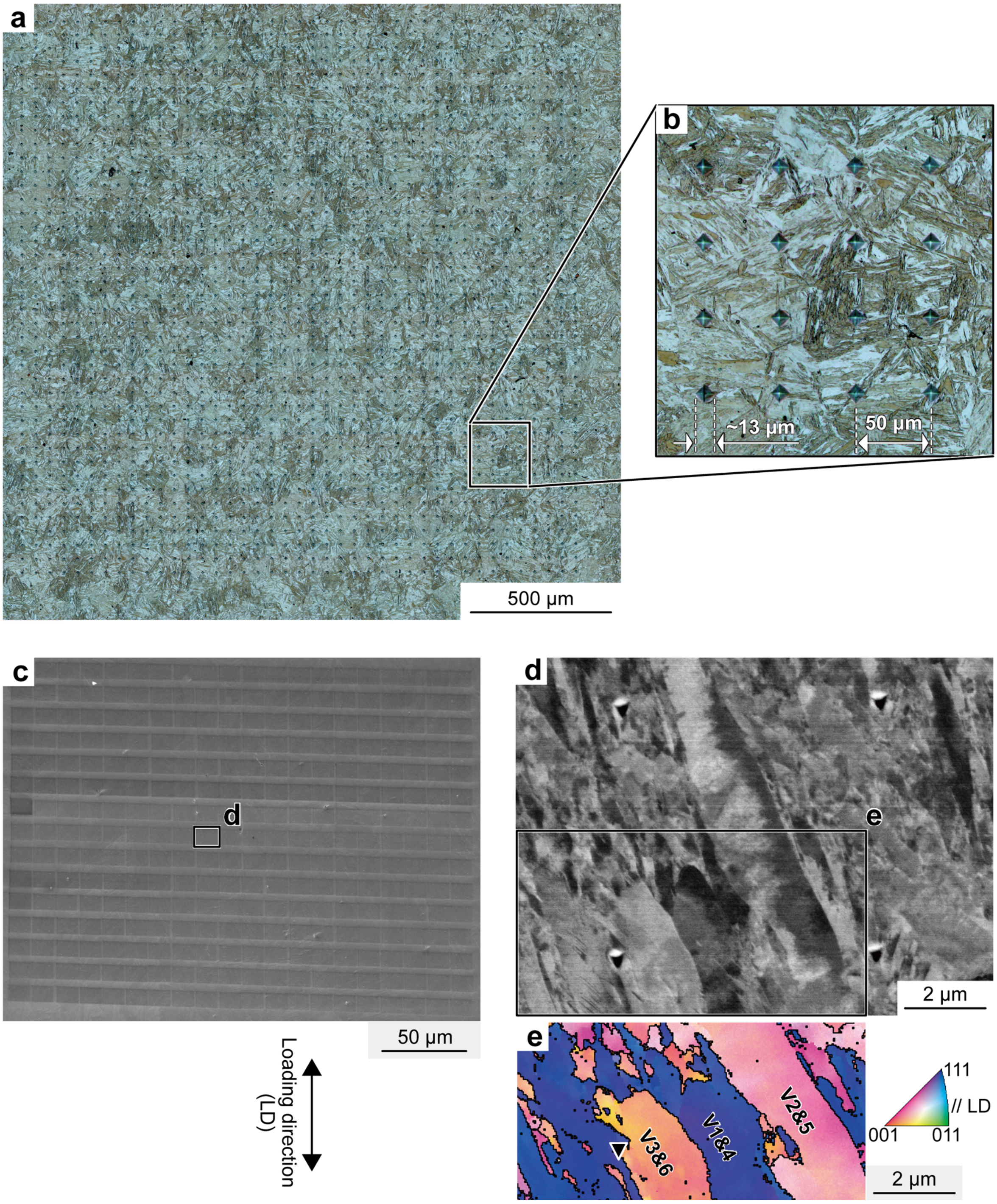


**Figure S9: Overview of multiscale statistical hardness measurements.** (a) Optical microscopy image of the area where 1681 points of Vickers hardness measurements were performed (the non-deformed specimen). (b) Enlarged view of (a); the measurement interval and size of the Vickers indentation were 50 μm and ~13 μm, respectively. (c) SEM image of the area where 1200 points of nanoindentation measurements were performed (the pre-fatigued + coaxing specimen). (d) Enlarged BSE image of (c); the measurement interval and size of the nanoindentation were 7 μm and ~300 nm, respectively. (e) EBSD orientation map corresponding to the black rectangle in (d).

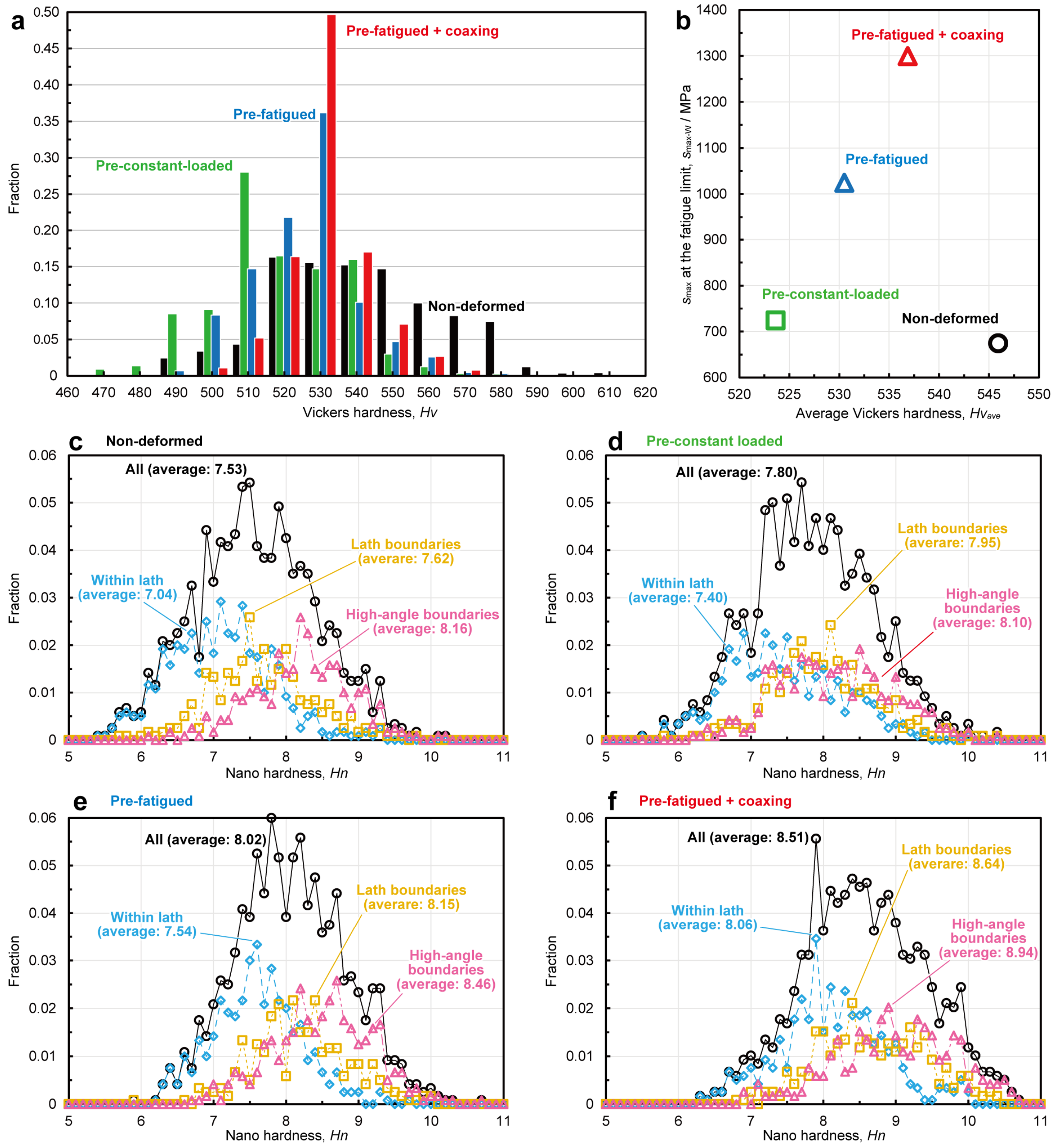


**Fig.S10: Results of multiscale statistical hardness measurements.** (a) Histograms of the Vickers hardness distribution; black, green, blue, and red indicate the non-deformed, pre-constant-loaded, pre-fatigued, and pre-fatigued + coaxing specimens, respectively. (b) Relationship between the $s_{\text{max-W}}$ and the average Vickers hardness ($Hv_{\text{ave}}$) of each specimen. No correlation between the $s_{\text{max-W}}$ and $Hv_{\text{ave}}$ was confirmed. Histograms of the nano-hardness distribution: (c) non-deformed, (d) pre-constant-loaded, (e) pre-fatigued, and (f) pre-fatigued + coaxing specimens. In each figure, the histogram including all data (1200 points, black circle) was decomposed into the data within lath (blue diamond), on lath boundaries (orange square), and on high-angle GBs (pink triangle).

**Table S1: Summary of the neutron diffraction analysis.** Young's modulus ($E_{hkl}$), Poisson's ratio ($\nu_{hkl}$), and normalized integrated intensities in the LD ($I_{LD\text{-}hkl}$) and TD ($I_{TD\text{-}hkl}$) of each lattice plane.

| Lattice plane | Young's modulus, $E_{hkl}$ (GPa) | Poisson's ratio, $\nu_{hkl}$ | Normalized integrated intensity | |
|---|---|---|---|---|
| | | | Loading direction (LD), $I_{LD\text{-}hkl}$ | Transverse direction (TD), $I_{TD\text{-}hkl}$ |
| 110 | 220 | 0.20 | 0.53 | 0.08 |
| 200 | 164 | 0.29 | 0.04 | 0.20 |
| 211 | 217 | 0.29 | 0.17 | 0.31 |
| 310 | 183 | 0.26 | 0.10 | 0.15 |
| 222 | 250 | 0.27 | 0.02 | 0.11 |
| 123 | 228 | 0.29 | 0.15 | 0.16 |
